# Prospective Analysis of Perioperative Stress Response in Living Donor Liver Transplantation for Hepatitis B-Related Liver Disease

**DOI:** 10.3390/jcm14248970

**Published:** 2025-12-18

**Authors:** Adem Tuncer, Sami Akbulut, Tevfik Tolga Sahin, Basri Satilmis, Zeki Ogut, Yasin Dalda, Sezai Yilmaz

**Affiliations:** 1 Department of Surgery, Faculty of Medicine, Istanbul Aydin University, 34295 Istanbul, Turkey; 2 Department of Surgery and Liver Transplant Institute, Faculty of Medicine, Inonu University, 44280 Malatya, Turkey; 3 Department of Biochemistry, Faculty of Pharmacy, Inonu University, 44280 Malatya, Turkey; 4 Department of Surgery, Elazig Fethi Sekin Education and Research Hospital, 23280 Elazig, Turkey

**Keywords:** hepatitis B virus infection, living donor liver transplantation, living donor hepatectomy, surgical trauma, stress response, cytokines, immune homeostasis

## Abstract

**Background**: Liver transplantation is a life-saving procedure for patients with end-stage liver disease, yet the immunological consequences of surgical trauma in these patients are not fully understood. The liver plays a central role in immune regulation, and its dysfunction in HBV-related chronic liver disease may alter the systemic stress response to surgery. **Aim**: This study aims to evaluate the stress response to surgical trauma of patients undergoing living donor liver transplantation (LDLT) for HBV-related chronic liver disease in comparison to living liver donors (LLDs). **Methods**: This prospective study included 20 LDLT recipients with HBV infection and 20 LLDs who underwent living donor hepatectomy between August 2020 and February 2021. Specific biochemical markers (IL-1, IL-4, IL-6, IL-22, IFN-γ, TNF-α, TGF-β, GM-CSF, GLDH, and GalactB) were measured at designated intervals: preoperative day 0 (Preop), immediately after incision (Incision), post-hepatectomy (Hepatectomy), postoperative day 0 (POD0), POD1, and POD3 using enzyme-linked immunosorbent assay (ELISA). Routine hematological and biochemical parameters (WBC, HGB, PLT, RDW, MPV, PDW, AST, ALT, ALP, GGT, albumin, total bilirubin, plateletcrit, phosphorus, fibrinogen, and INR) were measured regularly at five predetermined times: Preop, POD0, POD1, POD2, and POD3. **Results**: Prior to LDLT, LDLT recipients had significantly lower levels of pro-inflammatory cytokines (IL-1, IL-6, TNF-α, IFN-γ) compared to LLDs (*p* < 0.05). However, following liver implantation, these cytokine levels increased significantly at POD0, POD1, and POD3 (*p* < 0.001). Specifically, IL-1 levels elevated from 0 in the preop period to 21.5 (97.5) in POD3, and IL-6 elevated from 0 in the preop period to 28.3 at POD3 (*p* = 0.056). Similarly, TNF-α and IFN-γ levels exhibited significant upward trends (*p* < 0.05). In contrast, cytokine levels in LLDs remained stable throughout the perioperative period, revealing no statistically significant variations (*p* > 0.05). Routine hematological and biochemical parameters demonstrated significant postoperative fluctuations in LDLT recipients, reflecting the metabolic and immune restoration process. **Conclusions**: These findings indicate that patients with HBV-related chronic liver disease exhibit a diminished stress response to trauma due to underlying immune dysregulation caused by chronic hepatic dysfunction. However, after LDLT, the stress response gradually normalizes, suggesting that liver transplantation not only restores hepatic function but also reestablishes immune homeostasis, potentially reducing infection risks and improving postoperative recovery. These findings emphasize the crucial role of the liver in regulating the body’s stress response to trauma and highlight the immunological benefits of LDLT in restoring immune homeostasis.

## 1. Introduction

Starzl et al. [1] performed the first successful deceased donor liver transplantation (DDLT) in 1967, establishing it as the most effective therapeutic option for patients with end-stage liver disease; acute liver failure; selected metabolic disorders; primary hepatic malignancies; and, in rare cases, metastatic liver tumors [2,3]. Currently, liver transplantation (LT) can be performed using grafts obtained from either deceased donors—after brain death or donation after circulatory death (DCD)—or living liver donors (LLDs) [3]. The first living donor liver transplantation (LDLT) was reported by Raia et al. [4] in 1988. Despite significant progress, the disparity between organ demand and supply remains a major global issue, especially in developing and underdeveloped countries where deceased-donor organ availability is limited. Consequently, many patients die while awaiting transplantation [2,3]. LDLT emerged as a practical strategy to overcome this shortage and has become the predominant mode of transplantation in countries with low deceased-donor donation rates, such as Japan, India, Egypt, and Turkey. In Turkey, LDLT now constitutes the majority of all liver transplants [2,3]. However, LDLT requires the surgical removal of a substantial hepatic segment from a healthy donor, which carries a small but real risk of morbidity and mortality. This complex procedure therefore demands a careful balance between recipient benefit and donor safety, supported by meticulous preoperative assessment, surgical precision, and postoperative management.

LT is a complex abdominal operation that elicits a profound systemic inflammatory and metabolic response. In the context of liver failure, these functions are profoundly compromised, predisposing patients to dysregulated inflammatory and metabolic responses following major surgery [5]. The liver plays a pivotal role in regulating systemic inflammatory responses and maintaining immune homeostasis, both of which are crucial for recovery following major surgical trauma. In patients with chronic liver disease, this regulatory capacity becomes impaired due to dysfunctional cytokine production and altered immune signaling. Consequently, the stress response may become either blunted or exaggerated, leading to immune exhaustion and secondary immunosuppression. Among the numerous mediators of the surgical stress cascade, interleukin-6 (IL-6) and C-reactive protein (CRP) are well-established acute-phase reactants that directly correlate with the intensity of the inflammatory response and systemic stress burden [6,7,8,9]. LT, unlike most other surgical interventions, triggers a particularly complex and prolonged systemic reaction due to factors such as ischemia–reperfusion injury, massive tissue manipulation, and the preexisting immunological dysfunction associated with end-stage liver disease [10]. Jin et al. [11], reported that morbidity following major hepatic resections ranges from 4.09% to 47.7%, while mortality varies between 0.24% and 9.7%, underscoring the inherent risks and the ongoing need for strategies to enhance surgical safety and postoperative outcomes.

Dr. Walter Bradford Cannon first conceptualized the trauma-induced stress response during World War I, observing that although many patients survived the initial injury, secondary physiological complications frequently led to delayed shock and death [12]. He emphasized that the first 24–48 h following major trauma are critical for survival, as this period determines whether the body successfully restores homeostasis or succumbs to systemic failure. Changes in bowel motility disrupts enteric microbial barrier and results in translocation of bacteria and their products [13]. These stimulate the hepatic Kupffer cells and cause a low grade systemic inflammation. A dysregulated inflammatory cascade and a superimposed septic complication results in augmented inflammatory response that overwhelms regulatory mechanisms. Sustained inflammatory response can results in immune exhaustion, multiple organ dysfunction, and ultimately death that is defined as trauma-induced stress response and catabolic syndrome, underscoring the importance of balanced immune activation and timely intervention [13]. However, excessive or prolonged cytokine release can precipitate a “cytokine storm,” a state characterized by loss of immune homeostasis, metabolic disruption, and circulatory collapse leading to high morbidity and mortality

Following major surgical trauma—such as LT or living donor hepatectomy (LDH)—the immune system orchestrates a complex cascade of local and systemic responses aimed at restoring homeostasis [6,8,14]. Disruption of natural defensive barriers renders the surgical site highly susceptible to microbial invasion. Tissue factors and danger-associated molecular patterns released during hepatic injury amplify pro-inflammatory signaling in an exponential manner, driving the systemic inflammatory response [6,8,14]. A well-regulated immune balance is essential to achieve effective pathogen clearance and tissue repair while preventing excessive inflammation that could culminate in hemodynamic instability and organ failure. Among the central mediators of this process, interleukin-1 (IL-1), interleukin-6 (IL-6), and tumor necrosis factor-α (TNF-α) serve as key cytokines that initiate and sustain inflammation, promote leukocyte recruitment, and support wound healing [15,16]. Warm Ischemia during donor hepatectomy and portocaval occlusion during anhepatic phase in the recipient augments ischemia–reperfusion injury in the graft that compromise the success of liver transplantation [5]. In the recent literature, specific biomarkers—including Interleukin-1 (IL-1), Interleukin-4 (IL-4), Interleukin-6 (IL-6), Interleukin-22 (IL-22), interferon-γ (IFN-γ), TNF-α, transforming growth factor-β (TGF-β), granulocyte–macrophage colony-stimulating factor (GM-CSF), glutamate dehydrogenase (GLDH), and β-galactosidase—have been employed as quantitative indicators of immune activation, suppression, and hepatocellular injury [11].

Neutrophils play a pivotal role in trauma-induced immune responses. Upon activation, neutrophils originating from the liver, bone marrow, and spleen migrate into the systemic circulation and accumulate at sites of tissue injury, where they release elastase and reactive oxygen species (ROS) to limit pathogen spread. They also form neutrophil extracellular traps (NETs), composed of chromatin fibers and antimicrobial proteins, which serve as a physical barrier against invading microorganisms [17]. However, during persistent or dysregulated inflammation, immature neutrophils accumulate in the bloodstream, impairing adaptive immune responses and increasing susceptibility to infections—a condition known as Persistent Inflammation, Immunosuppression, and Catabolism Syndrome (PICS) [18]. The innate and innate-like immune systems are critically involved in the preservation–reperfusion phase of LT [6]. Prolonged cold ischemia time and reperfusion injury markedly upregulate inflammatory cytokines within the hepatic graft, particularly in DDLT [19]. These ischemia-related inflammatory cascades compromise early graft function and contribute to postoperative complications [6,19]. In contrast, LDLT benefits from shorter ischemia times and improved graft viability, offering a valuable clinical model for studying stress-related immune modulation.

Understanding these mechanisms is essential for optimizing perioperative management and improving transplant outcomes. The Inonu University Liver Transplantation Institute, established in 2002, is one of the highest-volume centers globally, with over 4000 LT procedures performed to date. In Turkey, hepatitis B virus (HBV)-induced chronic liver disease remains the leading indication for LT [20,21]. Unlike previous studies, the present investigation prospectively compares time-dependent cytokine responses in LDLT recipients and healthy LLDs across six defined perioperative intervals. By characterizing immune trajectories associated with hepatic injury, ischemia–reperfusion stress, and regeneration, this study aims to elucidate how surgical trauma and chronic liver disease influence systemic immunity. We hypothesize that LDLT recipients exhibit a blunted preoperative stress response due to chronic immune dysfunction, which progressively normalizes following transplantation as immune homeostasis is restored.

## 2. Materials and Methods

### 2.1. Study Design, Setting, Ethical Approval, and Funding

This prospective analytical study was designed to evaluate and compare the stress response to surgical trauma in two well-defined cohorts: 20 LDLT recipients with HBV-related chronic liver disease and 20 healthy LLDs who underwent LDH procedure during the same period. The study aimed to elucidate the perioperative immune and biochemical dynamics associated with major hepatic surgery under differing physiological and immunological conditions. The study was conducted at the Inonu University Liver Transplantation Institute—one of the world’s largest high-volume centers—following the ethical principles outlined in the 1964 Declaration of Helsinki and its later amendments. Approval was obtained from the Inonu University Institutional Review Board (Approval No: 131, Date: 12 August 2020) and the Directorate of the Liver Transplant Institute (Document No: E.45139, Date: 14 July 2020). All participants provided written informed consent prior to enrollment. Funding for this investigation was provided by the Inonu University Scientific Research Projects Coordination Unit (Project No: TSA-2020-2291). The study’s financial independence was maintained throughout the research process, and no external commercial entities were involved in data collection, analysis, or interpretation.

### 2.2. Study Population and Sample Size Calculation

Two cohorts were prospectively included two cohorts: 20 LDLT recipients (case arm) with HBV-related end-stage liver disease who underwent LT at the Inonu University Liver Transplantation Institute between August 2020 and February 2021, and 20 healthy LLDs (control arm) who underwent LDH in the same timeframe. The sample size determination was based on IL-6 concentrations reported in the study by Lan et al. [7], given the cytokine’s established role as a reliable biomarker of systemic inflammatory and post-surgical stress responses. In this comparative study, researchers investigated the levels of IL-6 in individuals undergoing hepatectomy. The study compared the IL-6 levels in individuals with normal livers (living donor hepatectomies) to those with diseased livers (hepatectomies performed for hepatocellular carcinoma). Specifically, the sample size calculation was conducted using the IL-6 levels at 48 h following the operation. The results revealed that the IL-6 levels were significantly elevated in individuals with diseased livers (109.50 ± 89.90) compared to those with normal livers (25.81 ± 12.15). Using MedCalc software (MedCalc Software Ltd., Ostend, Belgium; https://www.medcalc.org; accessed on 1 January 2020) and adopting a two-tailed hypothesis test with α = 0.01 and a statistical power (1−β) = 0.95, the minimum required sample size per group was calculated to be 18. To enhance analytical robustness and compensate for potential data loss, 20 participants were ultimately included in each arm.

### 2.3. Inclusion and Exclusion Criteria

Elective LDLT recipients with HBV-related chronic liver disease and healthy individuals who underwent LDH during the same period were included in the study. Pediatric LT cases, procedures involving the Pringle maneuver during donor hepatectomy, operations complicated by severe intraoperative hemodynamic instability, and participants with autoimmune diseases and any preoperative renal disease were excluded. Individuals receiving chronic corticosteroid or nonsteroidal anti-inflammatory therapy and those who had acquired immunodeficiency before LDLT, as well as those who declined participation, were also excluded.

### 2.4. Study Parameters

#### 2.4.1. Demographic, Clinical and Surgical Characteristics of the Patients

Demographic, anthropometric, and clinical characteristics—including age, sex, weight, body mass index (BMI), blood type, and degree of donor–recipient relationship—were prospectively recorded for all LDLT recipients (*n* = 20) and LLDs (*n* = 20). Routine and specific biochemical parameters were also documented. The surgical techniques for LDLT and LDH followed the standardized procedures previously described by our group [22,23,24,25]. Anesthetic management, intraoperative fluid administration, and transfusion strategies were conducted in accordance with our institutional protocols [26,27]. Perioperative antibiotic prophylaxis was administered based on established guidelines [28,29].

#### 2.4.2. Basic Biochemical and Hematological Parameters

Peripheral venous blood samples were collected from both LDLT recipients and LLDs at the following time points: preoperative baseline (PreOp), two hours after surgery (POD0), postoperative day 1 (POD1), postoperative day 2 (POD2), and postoperative day 3 (POD3). Routine biochemical and hematological analyses included measurements of white blood cell (WBC) count, hemoglobin (HGB), platelet count (PLT), red blood cell distribution width (RDW), mean platelet volume (MPV), platelet distribution width (PDW), plateletcrit, alanine transaminase (ALT), aspartate transaminase (AST), total bilirubin, gamma-glutamyl transferase (GGT), alkaline phosphatase (ALP), lactate dehydrogenase (LDH), albumin, phosphorus, international normalized ratio (INR), and C-reactive protein (CRP). All analyses were performed in the central biochemistry laboratory using automated analyzers that were calibrated daily according to manufacturer standards.

#### 2.4.3. Specific Biochemical Parameters

For specific biochemical and immunological analyses, blood samples were obtained from LDLT recipients and LLDs at six predefined time points: preoperative baseline (PreOp), after skin incision (Incision), immediately after completion of LDH or recipient hepatectomy (Hepatectomy), two hours post-surgery (POD0), postoperative day 1 (POD1), and postoperative day 3 (POD3). Samples were promptly transported to the Hepatology Research Laboratory of the Inonu University Liver Transplantation Institute. Serum was separated by centrifugation at 2000 rpm for 10 min at 4 °C, aliquoted, and stored at −80 °C until analysis. Cytokine and enzyme quantification included interleukin-1 (IL-1), interleukin-4 (IL-4), interleukin-6 (IL-6), interleukin-22 (IL-22), interferon-γ (IFN-γ), tumor necrosis factor-α (TNF-α), granulocyte–macrophage colony-stimulating factor (GM-CSF), transforming growth factor-β (TGF-β), glutamate dehydrogenase (GLDH), and β-galactosidase (GalactB). All assays were conducted using commercially available sandwich ELISA kits (Bioassay Technology Laboratory, Shanghai, China) according to the manufacturer’s protocols. The sensitivity (limit of detection, LOD) ranged from 0.1 to 10 pg/mL. All measurements were performed in duplicate, and laboratory personnel were blinded to the participant group allocation throughout the analyses.

### 2.5. Basic Immunosuppressive Protocol

The immunosuppressive treatment protocol implemented at our institute consists of induction with 500 mg of methylprednisolone, administered once the hepatic artery anastomosis is completed and hemostasis is secured. High-dose steroids are tapered over the first 10 days, while a fixed daily dose of 5 mg dexamethasone is maintained until three months after transplantation. Mycophenolate mofetil (MMF) is initiated at 500–1000 mg twice daily. Tacrolimus therapy begins on postoperative day 3. Steroids are discontinued by the third postoperative month, and MMF is withdrawn at six months. When renal dysfunction is present during transplantation, tacrolimus initiation is postponed until the first postoperative week, after renal function improves. In such situations, Basiliximab therapy is initiated on postoperative day 1 and administered weekly. If renal function does not recover and creatinine levels remain above 2 mg/dL, either tacrolimus-free immunosuppressive regimens or low-dose, sustained-release tacrolimus combined with MMF are employed. Importantly, none of the patients included in the present study had hepatorenal syndrome or perioperative renal impairment.

### 2.6. Statistical Analyses

Statistical analyses were performed using the Statistical Package for the Social Sciences (SPSS) version 25.0 (IBM Corp., Armonk, NY, USA). The distribution of continuous variables was assessed using the Shapiro–Wilk test. The results of the Shapiro–Wilk test are presented in the Supplementary Table S1. In summary, none of the continuous variables exhibited a normal distribution, and their values were expressed as median and interquartile range (IQR; Q3-Q1). Categorical variables were presented as counts and percentages. Comparisons of categorical variables between groups were made using the chi-square test, and between-group comparisons of continuous variables were evaluated using the Mann–Whitney U test or Student’s *t*-test, as appropriate. Longitudinal within-group changes in biochemical and cytokine levels were analyzed using the nonparametric Friedman test. When significant differences were detected, pairwise comparisons were performed using the Wilcoxon signed-rank test with Bonferroni correction to control for Type I error inflation. All analyses were conducted on complete-case datasets, as no missing data were observed during the study period. A two-tailed *p*-value < 0.05 was considered statistically significant.

## 3. Results

### 3.1. Comparison of LDLT Recipients and LLDs Groups

The demographic and specific biochemical parameters of the study recipients and donors are summarized in Table 1 and the basal characteristics of the study groups are given in the Supplementary Table S2. In the LDLT recipient group, 18 (90%) were male and 2 (10%) were female, whereas in the LLDs group, there were 13 males (65%) and 7 females (35%). There was no difference in terms of distribution of gender among the LDLT recipient and LLDs groups (*p* = 0.058). The median age of the recipients was 53.5 years (IQR = 11.7), ranging from 43 to 63 years, while the age of the donors ranged from 18 to 43 years (median = 28, IQR = 9.2). As expected, the median age of the recipients was significantly higher than that of the LLDs (*p* < 0.001). Furthermore, when we analyzed the age groups of the recipients, there were only 4 patients who were 60 years or older. No statistically significant difference was detected between the LDLT recipients (median = 25.7, IQR = 6.7) and LLDs (median = 23.8, IQR = 5.9) in terms of BMI (*p* = 0.253).

Statistically significant differences were observed between LDLT recipients and LLD groups in terms of cytokine levels measured at preop, incision, hepatectomy, POD0, POD1, and POD3. There was a significant difference in serum levels of hepatoprotective cytokine IL-22. On the other hand, GLDH and GalactB levels showed significant differences, which reflect hepatocyte regeneration. The levels of IL-1, TNF-α, IFN-γ, GM-CSF, IL-22, IL-4, GLDH, and GalactB were higher in the LLDs groups across all measured time points (*p* < 0.05). However, while IL-6 and TGF-β levels were significantly higher in the LLDs group at preop, incision, hepatectomy, POD0, and POD1, no statistically significant difference was found between the LLDs and LDLT recipients at POD3 (IL-6, *p* = 0.056; TGF-β, *p* = 0.157).

Routine biochemical parameters assessing the indirect reflections of the stress response to surgical trauma in LDLT recipients and LLDs were summarized in Table 2. Significantly higher levels (*p* < 0.05) of HGB, PLT, plateletcrit, and fibrinogen were found in the LLDs group at preop, POD0, POD1, and POD3 (*p* < 0.05). On the other hand, the recipient group displayed significantly higher RDW and MPV (POD2 borderline), ALT, and INR levels at preop, incision, hepatectomy, POD0, POD1, and POD3 time points in comparison to the donor group (*p* < 0.05). The LDLT recipients had higher levels of AST, GGT, and total bilirubin at preop, POD0, and POD1 time points, which were statistically significant compared to the do LLDs group (*p* < 0.05). However, these differences between the groups were no longer observed at POD2 or POD3 (*p* > 0.05). Albumin levels were significantly higher in the LLDs group preoperatively, on POD0, and POD1 (*p* < 0.001). On the other hand, there was no difference between the groups on POD2 and POD3 time intervals.

### 3.2. Intragroup Comparisons

#### 3.2.1. Intragroup Comparison in the LDLT Recipients Group

**Figure 1:**
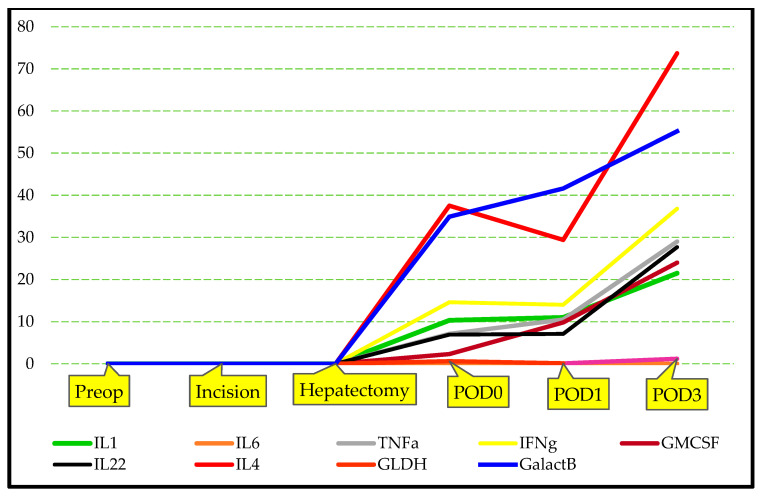
Graphical illustration of changes in specific blood cytokine levels of LDLT recipients group.

**Figure 2:**
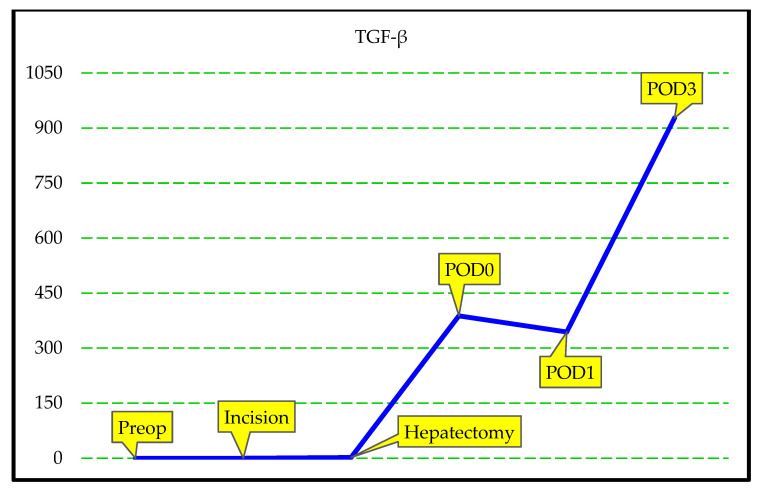
Graphical illustration of changes in specific blood TGF-β levels of LDLT recipients group.

The specific biochemical parameters measured at preop, hepatectomy, POD0, POD1, and POD3 time points were summarized in Table 3. The values of IL-1, IL-6, TNF-α, IFN-γ, GM-CSF, IL-22, IL-4, TGF-β, GLDH, and GalactB were close to 0 at the preop, incision, and hepatectomy periods. However, following liver implantation, all cytokine values increased gradually over time, and this increase was statistically significant (Figure 1 and Figure 2 [*p* < 0.001 for all cytokines; Friedman test]).

A statistically significant difference was identified in serum levels of IL-1 measured at preop and POD0 (*p* = 0.001), POD1 (*p* = 0.001), and POD3 (*p* = 0.001); between the IL-1 value at incision and POD0, POD1, and POD3; between the IL-1 value at hepatectomy and POD0, POD1, and POD3; and between the IL-1 value at POD0 and the IL-1 value at POD3. At POD1 and POD3, we observed a similar difference (Table 3).

A statistically significant difference was identified between the IL-6 levels at preop and POD0 (*p* = 0.028), POD1 (*p* = 0.028), and POD3 (*p* = 0.012); between IL-6 levels at incision and POD0, POD1, and POD3; and between IL-6 values at hepatectomy and POD0, POD1, and POD3. The IL-6 values at POD0 and POD3 showed a statistically significant difference (Table 3).

A statistically significant difference was discovered between the TNF-α values at preop and incision (*p* = 0.043), hepatectomy (*p* = 0.018), POD0 (*p* = 0.001), POD1 (*p* = 0.003), and POD3 (*p* = 0.001); between TNF-α at incision and hepatectomy, POD0, and POD1 and POD3; between the TNF-α value at hepatectomy and POD0, POD1, and POD3; between the TNF-α value at POD0 and POD3. We also observed a similar difference at POD1 and POD3 (Table 3).

A statistically significant difference was observed between the IFN-γ value at preop and hepatectomy (*p* = 0.046), POD0 (*p* = 0.002), POD1 (*p* = 0.002), and POD3 (*p* < 0.001); between the IFN-γ value at incision and POD0, POD1, and POD 3; between the IFN-γ value at hepatectomy and POD0, POD1, and POD3; and between the IFN-γ value at POD0 and POD3. We observed a similar difference at POD1 and POD3 (Table 3).

A statistically significant difference was identified between the GM-CSF values at preop and hepatectomy (*p* = 0.018), POD0 (*p* = 0.003), POD1 (*p* = 0.002), and POD3 (*p* < 0.001); between GM-CSF values at incision and POD0, POD1, and POD3; between GM-CSF value at hepatectomy and POD0, POD1, and POD3; and between GM-CSF value at POD0 and POD3. POD1 and POD3 also showed a similar difference (Table 3).

A statistically significant difference was detected between the IL-22 values at preop and hepatectomy (*p* = 0.050), POD0 (*p* = 0.003), POD1 (*p* = 0.002), and POD3 (*p* = 0.001); between IL-22 values at incision and POD0, POD1, and POD3; between IL-22 values at hepatectomy and POD0, POD1, and POD3; and between IL-22 values at POD0 and POD3. We also observed a similar difference between POD1 and POD3 (Table 3).

A statistically significant difference was observed between the IL-4 values at preop and hepatectomy (*p* = 0.018), POD0 (*p* = 0.001), POD1 (*p* = 0.001), and POD3 (*p* < 0.001); between the IL-4 value at incision and POD0, POD1, and POD3; between the IL-4 value at hepatectomy and POD0, POD1, and POD3; and between the IL-4 value at POD0 and POD3. We also observed a similar difference between POD1 and POD3 (Table 3).

We observed a statistically significant difference between the TGF-β value at preop and POD0 (*p* < 0.001) and POD1 (*p* < 0.001); between the TGF-β value at incision and POD0, POD1, and POD3; between the TGF-β value at hepatectomy and POD0, POD1, and POD3; and between the TGF-β value at POD0 and POD3. We also observed a similar difference at POD1 and POD3 (Table 3).

Also, a significant difference existed in the GLDH levels at preop compared to POD0 (*p* = 0.004), POD1 (*p* = 0.008), and POD3 (*p* = 0.001); between the GLDH levels at the incision compared to hepatectomy, POD0, POD1, and POD3; between the GLDH levels at the hepatectomy compared to POD0, POD1, and POD3; and between GLDH levels at POD0 and GLDH at POD3 (Table 3).

A significant difference was noted between the GalactB levels at preop compared to POD0 (*p* = 0.005), POD1 (*p* = 0.004), and POD3 (*p* = 0.002); between the GLDH levels at the incision compared to POD0, POD1, and POD3; between the GLDH levels at the hepatectomy compared to POD0, POD1, and POD3; and between the GalactB levels at POD0 and GalactB at POD3 (Table 3).

A significant correlation was demonstrated among the preoperative serum levels of IL-1, IL-6, TNF-α, IFN-γ, GM-CSF, IL-22, IL-4, TGF-β, GLDH, and GalactB (all *p* < 0.001). Detailed correlation results are presented in Supplementary Table S3 as a correlation matrix of preoperative cytokine levels. Furthermore, no association was identified between serum concentrations of IL-1, IL-6, TNF-α, IFN-γ, GM-CSF, IL-22, IL-4, TGF-β, GLDH, and GalactB measured preoperatively, on postoperative POD0, or on POD1, and the MELD scores of the patients. 

#### 3.2.2. Intragroup Comparison in LLDs Group

**Figure 3:**
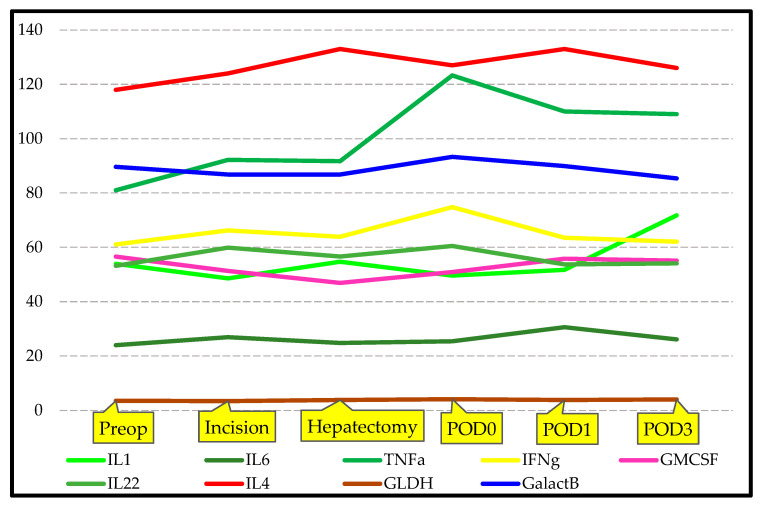
Graphical illustration of changes in specific blood cytokine levels of LLDs group.

**Figure 4:**
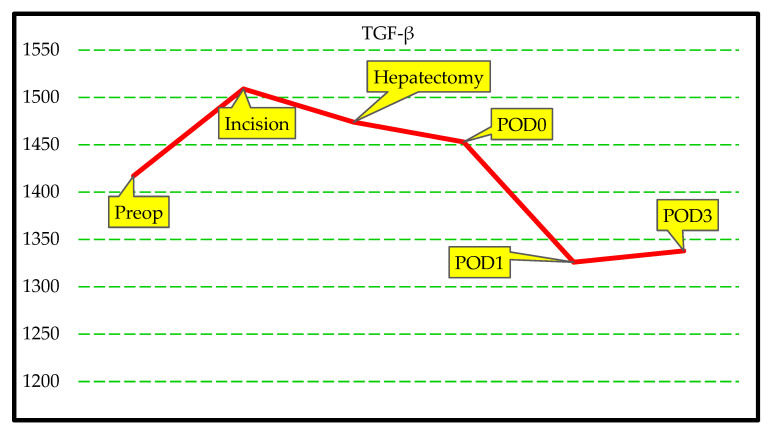
Graphical illustration of changes in specific blood TGF-β levels of LLDs group.

The serum IL-1, IL-6, TNF-α, IFN-γ, GM-CSF, IL-22, IL-4, TGF-β, GLDH, and GalactB values in the LLDs group measured in the preop, incision, hepatectomy, POD0, POD1, and POD3 intervals are summarized in Table 4. There was no statistically significant difference in the levels of IL-1, IL-6, TNF-α, IFN-γ, GM-CSF, IL-22, IL-4, TGF-β, GLDH, and GalactB at the defined time points. The results of the analyses are summarized in Table 4, Figure 3 and Figure 4. These results suggest that the cytokine markers of response to surgical trauma do not change significantly in healthy LLDs.

### 3.3. Comparison of Temporal Cytokine and Biochemical Marker Profiles Between LLDs and LDLT Recipients

Supplementary Figures S1–S10 illustrate the temporal trajectories of IL-1, IL-6, TNF-α, IFN-γ, GM-CSF, IL-22, IL-4, TGF-β, GLDH, and GalactB, measured at six perioperative time points in LLDs and LDLT recipients. Across all markers, repeated-measures analyses demonstrated significant between-group differences, with LLDs consistently exhibiting higher perioperative cytokine and chemokine responses, whereas LDLT recipients showed markedly attenuated patterns. Each figure visualizes these group-specific temporal dynamics, highlighting robust statistical significance for IL-1 (F = 25.3), IL-6 (F = 23.1), TNF-α (F = 15.3), IFN-γ (F = 14.1), GM-CSF (F = 15.4), IL-22 (F = 18.9), IL-4 (F = 29.3), TGF-β (F = 23.7), GLDH (F = 27.7), and GalactB (F = 5.8), thereby confirming distinct immunological profiles between the two groups over time.

## 4. Discussion

The systemic inflammatory response to trauma is a crucial physiological mechanism that prevents infection and provides the necessary machinery for tissue repair; however, this response differs significantly between LLDs and LDLT recipients due to the underlying liver condition and surgical extent [30]. In surgical procedures, including LT, this response is influenced by various factors such as the extent of surgery, ischemia–reperfusion injury, and the immune status of the patient [30,31]. The present study aimed to investigate the cytokine and biomarker responses in LLDs and LDLT recipients, delineating the key immunological differences between these two groups. The principal biomarkers identified in the present study—GLDH, GalactB, IL-6, TNF-α, TGF-β, IFN-γ, IL-22, GM-CSF, and IL-4—were classified according to their functional roles during surgical trauma adaptation into pro-inflammatory, anti-inflammatory, and regenerative categories. Graphical evaluation of the temporal fluctuations of these biomarkers in LDLT recipients and LLDs provided important insights into their dynamic roles within the context of transplantation. The present study demonstrated a notable pattern: overall pro-inflammatory, anti-inflammatory, and regenerative stimuli were markedly reduced in LDLT recipients, and this trend remained consistent across all time points. Conversely, all cytokines and chemokines evaluated in the present study showed a clear increase following implantation of the new liver.

GLDH, a mitochondrial enzyme, was notably elevated in LLDs preoperatively and peaked postoperatively, which supports the findings that associate GLDH with hepatic stress and ischemia–reperfusion injury [32]. This suggests that GLDH is a liver-specific marker and it is more efficient than conventional transaminases, making it a potential candidate for early detection of hepatic injury in LT settings [33,34]. The temporal changes in GLDH levels highlight the immediate postoperative surge in LLDs, whereas LDLT recipients exhibited a more gradual increase, reflecting delayed hepatic stress response and recovery patterns in the transplanted liver. In LDLT recipients, GLDH levels rose rapidly following the implantation of a healthy liver, whereas in LLDs, despite hepatectomy, GLDH showed a moderate increase before returning to baseline. This suggests that the surge in LDLT recipients reflects the active response of the implanted healthy liver to surgical stress. On the contrary, the transient rise in LLDs indicates a moderate adaptation to surgical stress to surgical trauma.

GalactB is a marker for ischemia–reperfusion injury known for its high sensitivity, and it showed a progressive postoperative increase in LDLT recipients, contrasting with its fluctuating levels in LLDs, which highlights the differences in regenerative and stress responses [22]. Given its early elevation compared to conventional liver enzymes, GalactB has potential as a biomarker for detecting ischemia–reperfusion injury at an earlier stage [35,36,37]. These results reinforce prior findings that support GalactB’s utility in early hepatic stress evaluation and suggest its inclusion in post-transplant biomarker panels. The progressive rise in GalactB levels strongly correlates with hepatocellular stress and subsequent recovery, emphasizing its potential as an early biomarker for liver function restoration [38,39,40]. The changes in GalactB closely resemble those seen in GLDH, reinforcing the different stress responses between LDLT recipients and LLDs.

It is a known fact that there is an immune dysfunction associated with decompensated cirrhosis [38]. Furthermore, the levels of pro-inflammatory cytokines such as IL-6 and TNF were reduced in patients with acute-on-chronic liver failure [39,40,41]. Usually, there is an increased systemic inflammation in chronic liver failure and compensated cirrhosis [38], but we found that even the pro-inflammatory stimuli were reduced in LDLT recipient. Furthermore, there was no correlation between the MELD scores and the levels of cytokines and other chemokines, indicating that disease severity did not influence the levels of the parameters investigated in our study. In additions, the levels of the cytokines and chemokines investigated in our study correlated with each other, which validated the accuracy of our results. In our opinion, HBV infection of our recipients may be responsible for the blunted inflammatory response. It has been demonstrated that active HBV infection induces alterations in the JAK/STAT pathway and the activity of matrix metalloproteinases, leading to immune dysfunction within the liver microenvironment [42].

Cytokines such as IL-6 and TNF-α are pivotal in modulating immune activation following LT [43]. In our study, IL-6 levels in LLDs exhibited a rapid postoperative rise, reflecting robust innate immune activation, whereas in LDLT recipients, the delayed peak suggests impaired Kupffer cell function and immune exhaustion in patients with chronic hepatic dysfunction. This distinction underscores the differential immune responses between healthy donors and transplant recipients, with IL-6 serving as a critical mediator of both early inflammation and liver regeneration. These findings are consistent with Lan et al. [7], who observed similar IL-6 elevation patterns in liver resection patients and demonstrated a correlation between cytokine fluctuations and hepatic regeneration. IL-6 also plays a key role in hepatocyte proliferation by activating the STAT3 signaling pathway, which supports liver regeneration [44]. The post-transplant increase in LDLT recipients underscores its significance in hepatic recovery after major surgical stress. The IL-6 pattern follows a trajectory similar to GLDH, indicating a close relationship between inflammation and hepatic stress response. Additionally, TNF-α exhibited distinct trends, where LLDs demonstrated a sharp increase post-hepatectomy followed by a return to baseline, whereas LDLT recipients experienced a rapid elevation post-implantation of a healthy liver. This pattern aligns with the role of TNF-α as a key mediator of M1 macrophage activation, which is associated with acute inflammatory responses and tissue damage repair. The sharp rise in cytokine levels among LLDs suggests an immediate but well-regulated inflammatory response, while the sustained elevation in LDLT recipients indicates prolonged immune activation due to the newly implanted liver graft [45,46]. Our results support the fact that TNF-α serves as a highly specific indicator of trauma response, particularly in distinguishing the initial inflammatory reaction in LLDs from the transplant-related immune activation in LDLT recipients. According to the present findings, LLDs serving as healthy controls had higher preoperative levels of the cytokines and chemokines investigated in our study. This may suggest a role for psychological stress [47] related to the LLD procedure, given that blood samples were obtained immediately before the operation.

TGF-β, a critical regulator of fibrosis and tissue remodeling [48], showed a unique trajectory in our study. While LLDs exhibited high preoperative levels due to normal regenerative signaling, LDLT recipients demonstrated a gradual postoperative increase. This pattern suggests that TGF-β monitoring could serve as an indicator of long-term graft adaptation and fibrosis risk, making it a key biomarker for post-transplant monitoring [45,49]. In the recipients, the TGF-β levels exhibited a sharp increase following liver implantation, whereas in LLDs, an initial rise was followed by a dramatic decline, even dropping below preoperative levels. Considering the preoperative stress factors experienced by LLDs, this outcome is understandable. We believe that if preoperative measurements had been taken days before the surgery, the true extent of the postoperative decline in LLDs would have been more apparent. In contrast, the response observed in LDLT recipients was the exact opposite of LLDs. The removal of the diseased liver, which had chronically low TGF-β production, allowed the newly implanted healthy liver to display a full stress response, revealing the true capacity of TGF-β as an indicator of surgical trauma adaptation Although the number of studies is not enough, some studies show TGF levels were associated with effector cell apoptosis and the resultant operational tolerance [50]. The correlation between the levels of TGF-β and the alloimmune response in liver grafts needs further research.

IFN-γ, an essential cytokine in modulating immune defense mechanisms, was significantly lower in LDLT recipients than in LLDs. Given that IFN-γ plays a role in viral immunity and macrophage activation, this observation suggests impaired immune responses in transplant recipients, likely due to chronic immunosuppression and hepatic dysfunction [51]. Studies have shown that post-transplant IFN-γ levels may correlate with the risk of infections and immune tolerance [46,52]. In the present study, the changes in IFN-γ closely resemble those observed in GLDH, reinforcing the differential stress responses between LDLT recipients and LLDs.

IL-22 has been implicated in hepatocyte protection and regeneration following liver injury [46,53]. Our study found significantly higher IL-22 levels in LLDs postoperatively compared to LDLT recipients, suggesting a role in immediate liver repair mechanisms. The lower levels in recipients may indicate compromised regenerative signaling, contributing to prolonged recovery times and increased susceptibility to postoperative complications [53]. Our results show that the changes in IL-22 closely resemble those observed in GLDH, reinforcing the differential stress responses between LDLT recipients and LLDs.

GM-CSF plays a crucial role in leukocyte activation and differentiation [54]. Our study observed elevated GM-CSF levels in LLDs, reflecting an intact hematopoietic response postoperatively. However, the delayed increase in LDLT recipients suggests an impaired ability to recruit and activate immune cells, further supporting the hypothesis of immune dysfunction in LDLT recipients [55,56]. According to our results, the changes in GM-CSF closely resemble those observed in GLDH, reinforcing the differential stress responses between LDLT recipients and LLDs.

IL-4, a key regulator of anti-inflammatory responses and tissue remodeling, was significantly elevated in LLDs but only moderately increased in LDLT recipients. IL-4 is primarily involved in M2 macrophage activation, which promotes tissue repair and fibrosis regulation [46,57]. The observed differences suggest that LLDs may undergo a more rapid transition to an anti-inflammatory state post-hepatectomy, while in LDLT recipients, this process is less pronounced due to underlying chronic hepatic dysfunction and immunosuppression. This discrepancy aligns with the notion that chronic liver disease shifts immune polarization towards an immunosuppressive phenotype, reducing the effectiveness of anti-inflammatory pathways necessary for wound healing and regeneration [49,58,59]. In the results of the present study, the changes in IL-4 closely resemble those observed in GLDH, reinforcing the differential stress responses between LDLT recipients and LLDs.

IL-1, a key pro-inflammatory cytokine, plays a crucial role in the early activation of Kupffer cells and amplification of immune responses following surgical trauma [35,59]. In our study, IL-1 levels increased sharply postoperatively in LLDs, reflecting acute hepatic stress, while in LDLT recipients, the rise was more gradual. This delayed response aligns with the patterns observed in IL-6 and TNF-α, reinforcing the role of IL-1 in initiating early inflammation and liver regeneration. The rapid IL-1 elevation in LLDs suggests a robust hepatic immune response, whereas the slower increase in LDLT recipients likely reflects impaired macrophage activation due to chronic liver disease [45,57].

Our findings reveal distinct biomarker and cytokine response patterns between LLDs and LDLT recipients, underscoring the immunological disparities induced by transplantation, emphasizing the importance of tracking immune recovery post-transplantation to assess clinical progress and guide immunosuppressive therapy. LLDs exhibited a robust inflammatory reaction with immediate cytokine elevation, whereas LDLT recipients displayed a delayed, blunted immune response, likely due to chronic liver disease and immunosuppressive treatment. The identification of GLDH, GalactB, IL-6, TNF-α, TGF-β, IFN-γ, IL-22, GM-CSF, and IL-4 as key biomarkers underscores their potential for monitoring LDLT outcomes and optimizing post-transplant care. Future studies should focus on integrating biomarker-based approaches into clinical practice to enhance LT management and improve patient prognosis, particularly those that explore composite scoring systems or immune recovery indexes for stratifying prognosis and treatment response. Analyzing cytokine patterns may help differentiate between patients with an uneventful course and those with a complicated one.

Fibrinogen and INR fluctuations have been identified as indicators of the surgical stress response [51,52,53,54,55,56,57,58,59,60,61,62,63], particularly in LDLT and LDH procedures. Our findings demonstrate significant post-operative changes in these markers, with LLDs exhibiting higher fibrinogen levels than LDLT recipients. This suggests a more controlled coagulation response in LLDs, while the persistently altered INR levels in LDLT recipients may reflect the prolonged hemostatic adaptation required after LDLT. These observations highlight the potential utility of fibrinogen and INR as biomarkers for assessing post-transplant recovery; however, future research should examine how dynamic changes in these markers relate to long-term graft outcomes, infection rates, and survival.

Despite the administration of perioperative and postoperative steroids, our study demonstrated a rapid postoperative increase in cytokine levels, particularly IL-6 and TNF-α, in LDLT recipients. This finding differs from Zhong’s study [63], where steroid administration was associated with a blunted cytokine response in non-transplanted patients. This study is a meta-analysis that included 895 patients (451 patients using steroids versus 444 patients using placebo in the perioperative period during hepatectomy). The discrepancy may be attributed to the presence of a newly implanted liver in LDLT recipients, which generates a robust immune response despite immunosuppressive therapy. Additionally, the present findings contrast with those reported by Lan et al. [7], in which IL-6 levels were associated with ischemia–reperfusion injury without a corresponding increase in TNF-α. Their analysis included 7 LDHs compared with 7 hepatectomy liver cancer patients with underlying liver disease. Because those patients had sufficient hepatic reserve to tolerate hepatectomy, the present results differ substantially from those of Lan et al. [7]. The distinct cytokine patterns observed in our study highlight the unique inflammatory environment in LT, influenced by both the graft and the recipient’s immune status. Future studies should aim to delineate the precise effects of steroid therapy on the dynamic post-transplant cytokine response.

It is widely acknowledged that aging adversely impacts endocrine, metabolic, and immune responses to injury [64]. The endocrine response diminishes due to insulin resistance, lower levels of insulin-like growth factor-1 (IGF-1), and reduced growth hormone levels [65]. These factors collectively lead to sarcopenia and changes in adipose tissue composition. In elderly individuals, sarcopenia combined with other systemic conditions such as hypertension and coronary artery disease fosters a pro-inflammatory state, along with chronic inflammation, through alterations in cytokine and chemokine profiles [66,67]. Nevertheless, these alterations are more pronounced in individuals over the age of 65 [68]. In our study, only six patients were over the age of 55, and four patients were 60 years or older. We believe this sample size is insufficient to influence the results, therefore we do not consider age difference as a limitation. Furthermore, our primary objective was to evaluate the impact of transplanted partial liver grafts on the host’s response to sepsis. The liver’s role in systemic inflammatory response syndrome (SIRS) is a dynamic process [69]. It serves as both a target and an effector organ during sepsis. In other words, SIRS may have detrimental effects on liver functions [69]. Conversely, the liver is a major organ that filters blood from infectious agents and xenobiotics, thereby reducing an exaggerated inflammatory response and liver failure may exacerbate innate immune system mediated inflammatory response [69].

### Limitations

The present study has several limitations. First, it focused on short-term postoperative cytokine and biomarker changes without long-term follow-up data. This limits our ability to evaluate the prolonged immune response and potential complications such as chronic graft dysfunction or fibrosis. Second, although perioperative steroid administration was standardized, variations in individual immune responses and the potential impact of additional immunosuppressive treatments were not specifically analyzed. Lastly, while we compared our results with existing literature, including studies by Zhong [63] and Lan [7], these have shown that differences in patient populations and clinical settings may affect the generalizability of our findings. All recipients and donors in our study experienced an uneventful postoperative course; therefore, we could not evaluate whether temporal changes in our parameters could have predicted deviations from the normal clinical course. Additionally, due to the limited number of studies investigating surgical stress response in LT patients, further validation with prospective multicenter studies is necessary to strengthen the clinical relevance of our results. Moreover, the potential effects of psychological trauma on immune modulation post-transplantation remain unexplored. Psychological burden may contribute to delayed recovery or influence cytokine dynamics, especially in recipients undergoing complex interventions. This represents a significant gap in understanding that future research should address.

## 5. Conclusions

Our study shows the substantial differences in biomarker and cytokine responses between LLDs and LDLT recipients, emphasizing the immunological impact of LT. LLDs exhibited a robust inflammatory reaction with immediate cytokine elevation, whereas LDLT recipients displayed a delayed, blunted immune response, likely due to chronic liver disease and immunosuppressive state. The identification of GLDH, GalactB, IL-6, TNF-α, TGF-β, IFN-γ, IL-22, GM-CSF, and IL-4 as key biomarkers underscores their potential for monitoring LDLT outcomes and optimizing post-transplant care. In addition to their diagnostic value, routine parameters such as fibrinogen and INR may complement immunological markers in tracking surgical stress adaptation. Moreover, the incorporation of these biomarkers into composite immune recovery scores may enhance individualized immunosuppression strategies. Future studies should also explore the role of psychological stress in modulating postoperative immune responses, particularly in complex transplant settings.

## Figures and Tables

**Table 1:** Comparison of LLDs and Liver Recipients groups in terms of some demographic and specific cytokine levels.

Variables [Median (IQR)]	LLDs (*n* = 20)	LDLT Recipients (*n* = 20)	*p*
Age (yıl)	28.0 (9.2)	53.5 (11.7)	<0.001
Gender (Male)	13 (65)	18 (90)	0.058
BMI (kg/m^2^)	23.8 (5.9)	25.7 (6.7)	0.253
IL-1			
Preop	53.9 (117.9)	0 (0)	<0.001
Incision	48.6 (49.6)	0 (0)	<0.001
Hepatectomy	54.7 (122)	0 (9.3)	<0.001
POD0	49.6 (112)	10.3 (27.8)	<0.001
POD1	51.7 (48.2)	11.0 (45.1)	<0.001
POD3	71.8 (53.2)	21.5 (97.5)	0.006
IL-6			
Preop	24.0 (20.4)	0 (0)	<0.001
Incision	26.9 (18.1)	0 (0)	<0.001
Hepatectomy	24.8 (15.4)	0 (0)	<0.001
POD0	25.4 (20.5)	0 (11.7)	0.001
POD1	30.6 (21.5)	0 (18.4)	0.001
POD3	26.1 (21.3)	0 (28.3)	0.056
TNF-α			
Preop	81.0 (81.0)	0 (0)	<0.001
Incision	92.2 (93.5)	0 (0.4)	<0.001
Hepatectomy	91.7 (99.8)	0 (11)	<0.001
POD0	123 (120)	7.1 (77.0)	0.004
POD1	110 (124)	10.5 (95.2)	0.001
POD3	109 (89)	29.0 (153)	0.049
INF-γ			
Preop	61.1 (70.1)	0 (0)	<0.001
Incision	66.2 (63.1)	0 (0)	<0.001
Hepatectomy	63.9 (65.4)	0 (23.2)	<0.001
POD0	74.8 (71.1)	14.6 (54)	<0.001
POD1	63.5 (71.9)	14.0 (65.7)	<0.001
POD3	62.1 (26.8)	36.8 (106)	0.012
GM-CSF			
Preop	56.6 (86.4)	0 (0)	<0.001
Incision	51.3 (66.0)	0 (0)	<0.001
Hepatectomy	46.9 (119)	0 (14.6)	<0.001
POD0	50.9 (128.6)	2.3 (76.4)	0.003
POD1	55.8 (52.5)	9.8 (59.5)	0.001
POD3	55.1 (60.4)	24.0 (106)	0.020
IL-22			
Preop	53.2 (32.7)	0 (0)	<0.001
Incision	59.9 (29.3)	0 (0)	<0.001
Hepatectomy	56.6 (35.0)	0 (19.9)	<0.001
POD0	60.5 (46.6)	6.9 (39.4)	0.004
POD1	53.7 (34.8)	7.1 (44.7)	0.004
POD3	54.1 (35.3)	27.7 (68.3)	0.024
IL-4			
Preop	118 (111)	0 (0)	<0.001
Incision	124 (86.0)	0 (0)	<0.001
Hepatectomy	133 (76.5)	0 (39.5)	<0.001
POD0	127 (90.5)	37.5 (71.2)	<0.001
POD1	133 (115)	29.4 (90.3)	<0.001
POD3	125 (110)	73.7 (132)	0.011
TGF-β			
Preop	1418 (1566)	0 (26.5)	<0.001
Incision	1509 (992)	0 (90.0)	<0.001
Hepatectomy	1474 (1076)	2.0 (327)	<0.001
POD0	1454 (1182)	388 (1228)	0.009
POD1	1326 (939)	344 (1404)	0.006
POD3	1338 (1374)	928 (1977)	0.157
GLDH			
Preop	3.5 (2.1)	0 (0)	<0.001
Incision	3.4 (2.8)	0 (0)	<0.001
Hepatectomy	3.8 (1.9)	0 (1.4)	<0.001
POD0	4.1 (3.4)	0.6 (2.4)	<0.001
POD1	3.8 (2.9)	0.1 (3.6)	0.001
POD3	4.0 (2.4)	1.2 (4.7)	0.008
GalactB			
Preop	89.6 (78.0)	0 (5.4)	<0.001
Incision	86.8 (39.7)	0 (7.2)	<0.001
Hepatectomy	86.8 (39.3)	0 (32.9)	<0.001
POD0	93.3 (44.6)	34.9 (57.7)	0.002
POD1	89.9 (48.6)	41.6 (76.5)	0.010
POD3	85.4 (37.9)	55.2 (118)	0.043

**Table 2:** Comparison of LLDs and Liver Recipients groups in terms of routine hematological and biochemical parameters.

Variables [Median (IQR)]	LLDs (*n* = 20)	LT Recipients (*n* = 20)	*p*
WBC			
Preop	7.3 (2.1)	6.0 (3.0)	0.003
POD0	22.5 (6.9)	18.1 (7.2)	0.006
POD1	15.9 (6.6)	10.4 (5.3)	0.004
POD2	12.9 (4.2)	11.8 (11.2)	0.201
POD3	8.3 (2.9)	8.1 (8.0)	0.277
HGB			
Preop	15.4 (3.9)	13.5 (2.1)	0.011
POD0	14.0 (3.1)	11.6 (3.7)	<0.001
POD1	14.2 (3.3)	10.2 (2.7)	<0.001
POD2	13.8 (3.3)	9.0 (2.6)	<0.001
POD3	13.0 (3.6)	9.0 (3.2)	<0.001
PLT			
Preop	242 (40.7)	79 (59.2)	<0.001
POD0	266 (91.5)	93 (62.0)	<0.001
POD1	192 (81.5)	70 (49.2)	<0.001
POD2	183 (65)	50 (41.7)	<0.001
POD3	180 (52.2)	49 (39.0)	<0.001
RDW			
Preop	12.8 (1.2)	14.3 (1.6)	<0.001
POD0	12.7 (1.4)	14.7 (3.8)	<0.001
POD1	12.7 (1.5)	14.9 (3.3)	<0.001
POD2	12.7 (1.8)	15.7 (3.1)	<0.001
POD3	12.6 (1.5)	16.0 (3.9)	<0.001
MPV			
Preop	10.3 (1.3)	11.5 (1.3)	0.002
POD0	10.2 (1.2)	11.3 (1.3)	0.007
POD1	10.2 (1.2)	11.5 (1.1)	0.006
POD2	10.5 (1.8)	11.2 (1.0)	0.058
POD3	10.4 (1.0)	11.2 (1.0)	0.007
PDW			
Preop	11.9 (2.5)	14.3 (3.3)	0.003
POD0	11.5 (2.8)	12.3 (2.8)	0.033
POD1	11.5 (2.3)	12.6 (2.9)	0.116
POD2	11.9 (3.3)	13.3 (2.1)	0.178
POD3	11.9 (2.1)	12.2 (2.5)	0.180
AST			
Preop	19.0 (9.5)	60.5 (52.7)	<0.001
POD0	125 (95.0)	478 (326.2)	<0.001
POD1	161 (127.7)	356 (241.5)	<0.001
POD2	123 (88.0)	171 (84.7)	0.114
POD3	82 (36.2)	99 (41.7)	0.242
ALT			
Preop	21 (9.2)	39.5 (30.5)	<0.001
POD0	146 (57.0)	546 (256.2)	<0.001
POD1	179 (115.2)	477 (288.5)	<0.001
POD2	166 (168.2)	317 (120.5)	0.002
POD3	125 (109.7)	223 (83.0)	0.002
ALP			
Preop	66.5 (27.7)	112 (77.7)	<0.001
POD0	64.5 (25.0)	62.0 (41.2)	0.799
POD1	62.5 (26.2)	52.0 (24.0)	0.030
POD2	70.5 (38.7)	45.0 (19.0)	<0.001
POD3	78.0 (47.0)	45.0 (19.0)	<0.001
Albumin			
Preop	4.3 (0.52)	2.6 (0.90)	<0.001
POD0	3.5 (0.48)	2.25 (0.80)	<0.001
POD1	3.3 (0.40)	2.7 (0.68)	<0.001
POD2	3.3 (0.40)	3.0 (0.7)	0.108
POD3	3.3 (0.30)	3.2 (0.7)	0.841
GGT			
Preop	18.0 (13.0)	62.0 (65.7)	<0.001
POD0	21.5 (17.7)	42.0 (50.7)	0.003
POD1	26.5 (37.5)	40.0 (38.5)	0.021
POD2	26.5 (39.7)	33.0 (28.2)	0.211
POD3	51.0 (59.5)	40.5 (32.0)	0.547
Phosphorus			
Preop	3.4 (0.98)	3.2 (0.7)	0.369
POD0	3.6 (1.2)	4.1 (1.2)	0.013
POD1	2.9 (0.7)	3.3 (1.6)	0.277
POD2	2.3 (1.0)	2.6 (1.4)	0.006
POD3	2.6 (0.9)	3.1 (0.9)	0.003
Total Bilirubin			
Preop	0.6 (0.2)	1.6 (2.5)	<0.001
POD0	1.6 (0.6)	5.7 (3.4)	<0.001
POD1	2.1 (1.3)	4.8 (4.4)	<0.001
POD2	2.6 (2.0)	2.2 (4.0)	0.947
POD3	2.45 (1.6)	1.9 (2.2)	0.678
Plateletcrit			
Preop	0.2 (0.1)	0.1 (0.10)	<0.001
POD0	0.3 (0.1)	0.1 (0.02)	<0.001
POD1	0.2 (0.0)	0.1 (0.02)	<0.001
POD2	0.2 (0.0)	0.1 (0.10)	<0.001
POD3	0.2 (0.0)	0.1 (0.10)	<0.001
Fibrinogen			
POD0	196 (105)	100 (49)	<0.001
POD1	288 (205)	102 (47)	<0.001
POD2	347 (129)	132 (48)	<0.001
POD3	371 (109)	133 (53)	<0.001
INR			
Preop	1.0 (0.1)	1.40 (0.3)	<0.001
POD0	1.2 (0.2)	2.85 (1.2)	<0.001
POD1	1.5 (0.2)	2.45 (0.8)	<0.001
POD2	1.4 (0.2)	2.10 (0.6)	<0.001
POD3	1.3 (0.2)	1.55 (0.5)	0.002

ALP: Alkaline Phosphatase; ALT: Alanine Aminotransferase; AST: Aspartate Aminotransferase; BMI: Body Mass Index; CRP: C-Reactive Protein; GalactB: β-galactosidase; GGT: Gamma-Glutamyl Transferase; GLDH: Glutamate Dehydrogenase; GM-CSF: Granulocyte-Macrophage Colony-Stimulating Factor; HGB: Hemoglobin; IFN-γ: Interferon-γ; IL-1: Interleukin-1; IL-4: Interleukin-4; IL-6: Interleukin-6; IL-22: Interleukin-22; INR: International Normalized Ratio; MPV: Mean Platelet Volume; PDW: Platelet Distribution Width; PLT: Platelet Count; RDW: Red Cell Distribution Width; TGF-β: Transforming Growth Factor-β; TNF-α: Tumor Necrosis Factor-α; WBC: White Blood Cell.

**Table 3:** Changes in blood cytokine levels assessed at six separate periods in LDLT recipients throughout time.

	Preop	Incision	Hepatectomy	POD0	POD1	POD3
IL-1	0 (0) ^a.b.c^	0 (0) ^d.e.f^	0 (9.3) ^g.h.i^	10.3 (27.8) ^a.d.g.j^	11.0 (45.1) ^b.e.h.k^	21.5 (97.5) ^c.f.i.j.k^
IL-6	0 (0) ^a.b.c^	0 (0)	0 (0) ^d.e.f^	0 (11.7) ^a.d.g^	0 (18.4) ^b.e^	0 (28.3) ^c.f.g^
TNF-α	0 (0) ^a.b.c.d.e^	0 (0.4) ^a.f.g.h.i^	0 (11.0) ^b.f.j.k.l^	7.1 (77.0) ^c.g.j.m^	10.5 (95.2) ^d.h.k.n^	29.0 (152.7) ^e.i.l.m.n^
IFN-γ	0 (0) ^a.b.c.d^	0 (0) ^e.f.g^	0 (23.2) ^a.h.i.j^	14.6 (54.0) ^b.e.h.k^	14.0 (65.7) ^c.f.l^	36.8 (105.7) ^d.g.j.k.l^
GMCSF	0 (0) ^a.b.c.d^	0 (0) ^e.f.g^	0 (14.6) ^a.h.i.j^	2.3 (76.4) ^b.e.h.k^	9.8 (59.5) ^c.f.i.l^	24.0 (105.6) ^d.g.j.k.l^
IL-22	0 (0) ^a.b.c.d^	0 (0) ^e.f.g^	0 (19.9) ^a.h.i.j^	6.9 (39.4) ^b.e.h.k^	7.1 (44.7) ^c.f.i.l^	27.7 (68.3) ^d.g.j.k.l^
IL-4	0 (0) ^a.b.c.d^	0 (0) ^e.f.g^	0 (39.5) ^a.h.i.j^	37.5 (71.2) ^b.e.h.k^	29.4 (90.3) ^c.f.i.l^	73.7 (132.1) ^d.g.j.k.l^
TGF-β	0 (26.5) ^a.b.c^	0 (90.0) ^d.e.f^	2.0 (327.4) ^g.h.i^	388 (1228.3) ^a.d.g.j^	344 (1404.6) ^b.e.h.k^	928 (1977.7) ^c.f.i.j.k^
GLDH	0 (0) ^a.b.c^	0 (0) ^d.e.f.g^	0 (1.4) ^d.h.i.j^	0.6 (2.4) ^a.e.h.k^	0.1 (3.6) ^b.f.i^	1.2 (4.7) ^c.g.j.k^
GalactB	0 (5.4) ^a.b.c^	0 (7.2) ^d.e.f^	0 (32.9) ^g.h.i^	34.9 (57.7) ^a.d.g.j^	41.6 (76.5) ^b.e.h^	55.2 (118) ^c.f.i.j^

Friedman and Wilcoxon signed-rank tests were used to compare dependent groups and to determine the groups responsible for the differences, respectively. GalactB: β-galactosidase; GLDH: Glutamate dehydrogenase; GM-CSF: Granulocyte–macrophage colony-stimulating factor; IFN-γ: Interferon gamma; IL-1: Interleukin-1; IL-22: Interleukin-22; IL-4: Interleukin-4; IL-6: Interleukin-6; TGF-β: Transforming growth factor beta; TNF-α: Tumor necrosis factor alpha. IL-1 [a, b, c, d, e, g, h, i (*p* = 0.001); f (*p* < 0.001); j (*p* = 0.007)]; IL-6 [a, b, d, e (*p* = 0.028); c, f (*p* = 0.012); g (*p* = 0.036)]; TNF-α [a (*p* = 0.043); b (*p* = 0.018); c, e, g, i (*p* = 0.001); d (*p* = 0.003); f (*p* = 0.038); h, l (*p* = 0.005); j (*p* = 0.009); k (*p* = 0.028); m (*p* = 0.036); n (*p* = 0.016)]; INF-γ [a (*p* = 0.046); b, c, e, f, h, i (*p* = 0.002); d, g, j (*p* < 0.001); k (*p* = 0.011); l (*p* = 0.001)]; GMCSF [a (*p* = 0.018); b, e, h (*p* = 0.003); c, f (*p* = 0.002); d, g, j (*p* < 0.001); i (*p* = 0.015); k (*p* = 0.005); l (*p* = 0.001)]; IL-4 [a (*p* = 0.018); b, c, e, f, h, i (*p* = 0.001); d, g, j, k (*p* < 0.001); l (*p* = 0.002)]; IL-22 [a (*p* = 0.050); b, e (*p* = 0.003); c, f (*p* = 0.002); d, g, j (*p* = 0.001); h (*p* = 0.008); i (*p* = 0.006); k (*p* = 0.022); l (*p* = 0.013)]; TGF-β [a, b, c, d, e, f, g, h, i (*p* < 0.001); j (*p* = 0.005); k (*p* = 0.001)]; GLDH [a (*p* = 0.004); b, f (*p* = 0.008); c, e, g (*p* = 0.001); d (*p* = 0.028); h (*p* = 0.006); i (*p* = 0.017); j (*p* = 0.002); k (*p* = 0.041)]; GalactB [a (*p* = 0.005); b (*p* = 0.004); c, d (*p* = 0.002); e, i (*p* = 0.001); f (*p* < 0.001); g (*p* = 0.017); h (*p* = 0.015); j (*p* = 0.028)].

**Table 4:** Changes in blood cytokine levels assessed at six separate periods in LLDs throughout time.

	Preop	Incision	Hepatectomy	POD0	POD1	POD3	*p*
IL-1	53.9 (117.9)	48.6 (49.6)	54.7 (122.0)	49.6 (111.9)	51.7 (48.2)	71.8 (53.2)	0.983
IL-6	24.0 (20.4)	26.9 (18.1)	24.8 (15.4)	25.4 (20.5)	30.6 (21.5)	26.1 (21.3)	0.414
TNF-α	81.0 (81.0)	92.2 (93.5)	91.7 (99.8)	123.3 (120)	110 (125)	109 (89)	0.668
IFN-γ	61.1 (70.1)	66.2 (63.1)	63.9 (65.4)	74.8 (71.1)	63.5 (71.9)	62.1 (26.8)	0.91
GMCSF	56.6 (86.4)	51.3 (66.0)	46.9 (119.2)	50.9 (128.6)	55.8 (52.5)	55.1 (60.4)	0.995
IL-22	53.2 (32.7)	59.9 (29.3)	56.6 (35.0)	60.5 (46.6)	53.7 (34.8)	54.1 (35.3)	0.395
IL-4	118 (110)	124 (86)	133 (76.5)	127 (90.5)	133 (114)	126 (110)	0.324
TGF-β	1417 (1566)	1509 (992)	1474 (1075)	1453 (1182)	1326 (939)	1338 (1374.4)	0.916
GLDH	3.5 (2.1)	3.4 (2.8)	3.8 (1.9)	4.1 (3.4)	3.8 (2.9)	4.0 (2.4)	0.055
GalactB	89.6 (78.0)	86.8 (39.7)	86.8 (39.3)	93.3 (44.6)	89.9 (48.6)	85.4 (37.9)	0.216

## Data Availability

The datasets analyzed during the current study are available from the corresponding author on reasonable request.

## Supplementary Materials

The following supporting information can be downloaded at: https://www.mdpi.com/article/10.3390/jcm14248970/s1, Table S1. Shapiro–Wilk Normality Test results for some preoperative routine biochemical and cytokine Parameters; Table S2: The baseline (preoperative) characteristics of the LLD and LDLT Recipients groups; Table S3: Correlation Matrix of Preoperative Cytokine Levels; Figure S1: Temporal IL-1 Response in LLDs and LDLT Recipients Across Six Perioperative Time Points (F = 25.3, *p* < 0.001); Figure S2: Temporal IL-6 Response in LLDs and LDLT Recipients Across Six Perioperative Time Points (F = 23.1, *p* < 0.001); Figure S3: Temporal TNF-α Response in LLDs and LDLT Recipients Across Six Perioperative Time Points (F = 15.3, *p* < 0.001); Figure S4: Temporal IFN-ɣ Response in LLDs and LDLT Recipients Across Six Perioperative Time Points (F = 14.1, *p* = 0.001); Figure S5: Temporal GM-CSF Response in LLDs and LDLT Recipients Across Six Perioperative Time Points (F = 15.4, *p* < 0.001); Figure S6: Temporal IL-22 Response in LLDs and LDLT Recipients Across Six Perioperative Time Points (F = 18.9, *p* < 0.001); Figure S7: Temporal IL-4 Response in LLDs and LDLT Recipients Across Six Perioperative Time Points (F = 29.3, *p* < 0.001); Figure S8: Temporal TGF-β Response in LLDs and LDLT Recipients Across Six Perioperative Time Points (F = 23.7, *p* < 0.001); Figure S9: Temporal GLDH Response in LLDs and LDLT Recipients Across Six Perioperative Time Points (F = 27.7, *p* < 0.001); Figure S10: Temporal GalactB Response in LLDs and LDLT Recipients Across Six Perioperative Time Points (F = 5.8, *p* = 0.021).
