# Supplementary material for: Prospective Analysis of Perioperative Stress Response in Living Donor Liver Transplantation for Hepatitis B-Related Liver Disease
Source: J Clin Med. 2025 Dec 18;14(24):8970. doi: 10.3390/jcm14248970 (PMC12733402; doi:10.3390/jcm14248970)

Supplementary Table S1: Shapiro–Wilk Normality Test results for some preoperative routine biochemical and cytokine parameters

Parameters	df	Statistics	Sig
Age (years)	14	0.952	0.593
BMI (kg/m^2)	14	0.893	0.088
MELD	14	0.965	0.808
IL1 (Preop)	14	0.444	0.000
IL-6 (Preop)	14	0.438	0.000
TNF-α (Preop)	14	0.297	0.000
INF-γ (Preop)	14	0.447	0.000
GMCSF (Preop)	14	0.306	0.000
IL-22 (Preop)	14	0.310	0.000
IL-4 (Preop)	14	0.431	0.000
TGF-β (Preop)	14	0.536	0.000
GLDH (Preop)	14	0.477	0.000
GalactB (Preop)	14	0.536	0.000
WBC (Preop)	14	0.934	0.343
HGB (Preop)	14	0.913	0.174
PLT (Preop)	14	0.796	0.004
RDW (Preop)	14	0.730	0.001
MPV (Preop)	14	0.974	0.928
PDW (Preop)	14	0.981	0.980
AST (Preop)	14	0.514	0.000
ALT (Preop)	14	0.628	0.000
CRP (Preop)	14	0.799	0.005
GGT (Preop)	14	0.877	0.053
ALP (Preop)	14	0.895	0.096
Albumin (Preop)	14	0.919	0.008
Bilirubin (Preop)	14	0.970	0.371
Phosphorus (Preop)	14	0.416	0.000

ALP: Alkaline Phosphatase; ALT: Alanine Aminotransferase; AST: Aspartate Aminotransferase; BMI: Body Mass Index; CRP: C-Reactive Protein; GalactB: β-galactosidase; GGT: Gamma-Glutamyl Transferase; GLDH: Glutamate Dehydrogenase; GM-CSF: Granulocyte-Macrophage Colony-Stimulating Factor; HGB: Hemoglobin; IFN-γ: Interferon-γ; IL-1: Interleukin-1; IL-4: Interleukin-4; IL-6: Interleukin-6; IL-22: Interleukin-22; MELD: Model for End-Stage Liver Disease; MPV: Mean Platelet Volume; PDW: Platelet Distribution Width; PLT: Platelet Count; RDW: Red Cell Distribution Width; TGF-β: Transforming Growth Factor-β; TNF-α: Tumor Necrosis Factor-α; WBC: White Blood Cell

Supplementary Table S2. The baseline (preoperative) characteristics of the LLD and LDLT recipients groups.

Variables [Median(IQR)]	LLDs (n=20)	LDLT Recipients (n=20)	p
Age (years)	28.0 (9.2)	53.5 (11.7)	<0.001
Gender (Male)	13 (65)	18 (90)	0.058
BMI (kg/m^2)	23.8 (5.9)	25.7 (6.7)	0.253
IL-1	53.9 (117.9)	0 (0)	<0.001
IL-6	24.0 (20.4)	0 (0)	<0.001
TNF-α	81.0 (81.0)	0 (0)	<0.001
INF-γ	61.1 (70.1)	0 (0)	<0.001
GMCSF	56.6 (86.4)	0 (0)	<0.001
IL-22	53.2 (32.7)	0 (0)	<0.001
IL-4	118 (111)	0 (0)	<0.001
TGF-β	1418 (1566)	0 (26.5)	<0.001
GLDH	3.5 (2.1)	0 (0)	<0.001
GalactB	89.6 (78.0)	0 (5.4)	<0.001
WBC	7.3 (2.1)	6.0 (3.0)	0.003
HGB	15.4 (3.9)	13.5 (2.1)	0.011
PLT	242 (40.7)	79 (59.2)	<0.001
RDW	12.8 (1.2)	14.3 (1.6)	<0.001
MPV	10.3 (1.3)	11.5 (1.3)	0.002
PDW	11.9 (2.5)	14.3 (3.3)	0.003
AST	19.0 (9.5)	60.5 (52.7)	<0.001
ALT	21 (9.2)	39.5 (30.5)	<0.001
ALP	66.5 (27.7)	112 (77.7)	<0.001
Albumin	4.3 (0.52)	2.6 (0.90)	<0.001
GGT	18.0 (13.0)	62.0 (65.7)	<0.001
Phosphorus	3.4 (0.98)	3.2 (0.7)	0.369
Total Bilirubin	0.6 (0.2)	1.6 (2.5)	<0.001
Plateletcrit	0.2 (0.1)	0.1 (0.10)	<0.001
INR	1.0 (0.1)	1.40 (0.3)	<0.001

ALP: Alkaline Phosphatase; ALT: Alanine Aminotransferase; AST: Aspartate Aminotransferase; BMI: Body Mass Index; CRP: C-Reactive Protein; GalactB: β-galactosidase; GGT: Gamma-Glutamyl Transferase; GLDH: Glutamate Dehydrogenase; GM-CSF: Granulocyte-Macrophage Colony-Stimulating Factor; HGB: Hemoglobin; IFN-γ: Interferon-γ; IL-1: Interleukin-1; IL-4: Interleukin-4; IL-6: Interleukin-6; IL-22: Interleukin-22; INR: International Normalized Ratio; MPV: Mean Platelet Volume; PDW: Platelet Distribution Width; PLT: Platelet Count; RDW: Red Cell Distribution Width; TGF-β: Transforming Growth Factor-β; TNF-α: Tumor Necrosis Factor-α; WBC: White Blood Cell

Supplementary Table S3: Correlation Matrix of Preoperative Cytokine Levels

	**Statistics**	**IL-1**	**IL-6**	**TNF-α**	**GMCSF**	**IL-22**	**IL-4**	**TGF-β**	**GLDH**	**GalactB**
IL-1 (preop)	Pearson	1	.570	.607	.821	.772	.649	.730	.843	.757
	Sig		0.000	0.000	0.000	0.000	0.000	0.000	0.000	0.000
IL-6 (preop)	Pearson	.570	1	.859	.741	.639	.904	.838	.760	.850
	Sig	0.000		0.000	0.000	0.000	0.000	0.000	0.000	0.000
TNF-α (preop)	Pearson	.607	.859	1	.720	.740	.848	.890	.791	.789
	Sig	0.000	0.000		0.000	0.000	0.000	0.000	0.000	0.000
INF-γ (preop)	Pearson	.821	.741	.720	1	.554	.769	.796	.852	.834
	Sig	0.000	0.000	0.000		.000	.000	.000	.000	.000
GMCSF (preop)	Pearson	.772	.639	.740	.554	1	.686	.730	.787	.659
	Sig	0.000	0.000	0.000	0.000		0.000	0.000	0.000	0.000
IL-22 (preop)	Pearson	.649	.904	.848	.769	.686	1	.921	.833	.926
	Sig	0.000	0.000	0.000	0.000	0.000		0.000	0.000	0.000
IL-4 (preop)	Pearson	.730	.838	.890	.796	.730	.921	1	.878	.947
	Sig	.000	.000	.000	.000	.000	.000		0.000	0.000
TGF-β (preop)	Pearson	.843	.760	.791	.852	.787	.833	.878	1	.830
	Sig	0.000	0.000	0.000	0.000	0.000	0.000	0.000		0.000
GLDH (preop)	Pearson	.757	.850	.789	.834	.659	.926	.947	.830	1
	Sig	0.000	0.000	0.000	0.000	0.000	0.000	0.000	0.000	
GalactB (preop)	Pearson	.523	.650	.494	.640	.357	.685	.610	.534	.690
	Sig	0.000	0.000	0.000	0.000	0.000	0.000	0.000	0.000	0.000

GalactB: β-galactosidase; GLDH: Glutamate Dehydrogenase; GM-CSF: Granulocyte-Macrophage Colony-Stimulating Factor; IL-1: Interleukin-1; IL-4: Interleukin-4; IL-6: Interleukin-6; IL-22: Interleukin-22; TGF-β: Transforming Growth Factor-β; TNF-α: Tumor Necrosis Factor-α

Supplementary Figure S1: Temporal IL-1 Response in LLDs and LDLT Recipients Across Six Perioperative Time Points (F = 25.3, p < 0.001)

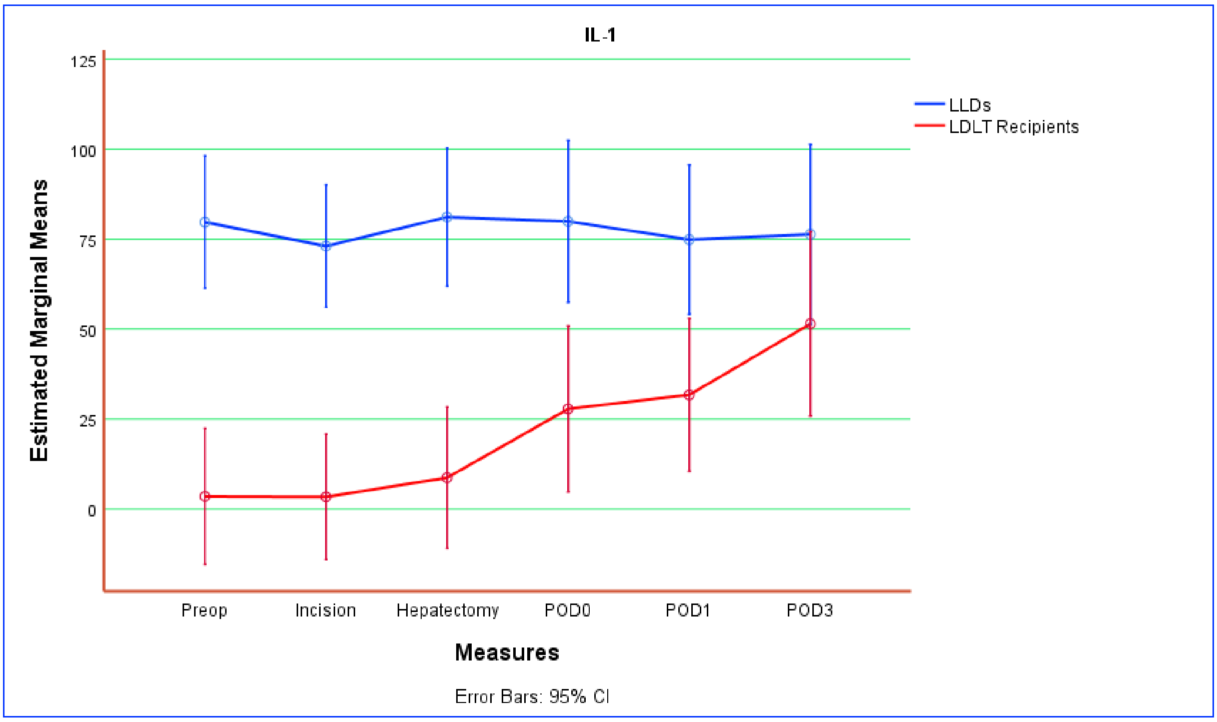

Supplementary Figure S2: Temporal IL-6 Response in LLDs and LDLT Recipients Across Six Perioperative Time Points (F = 23.1, p < 0.001)

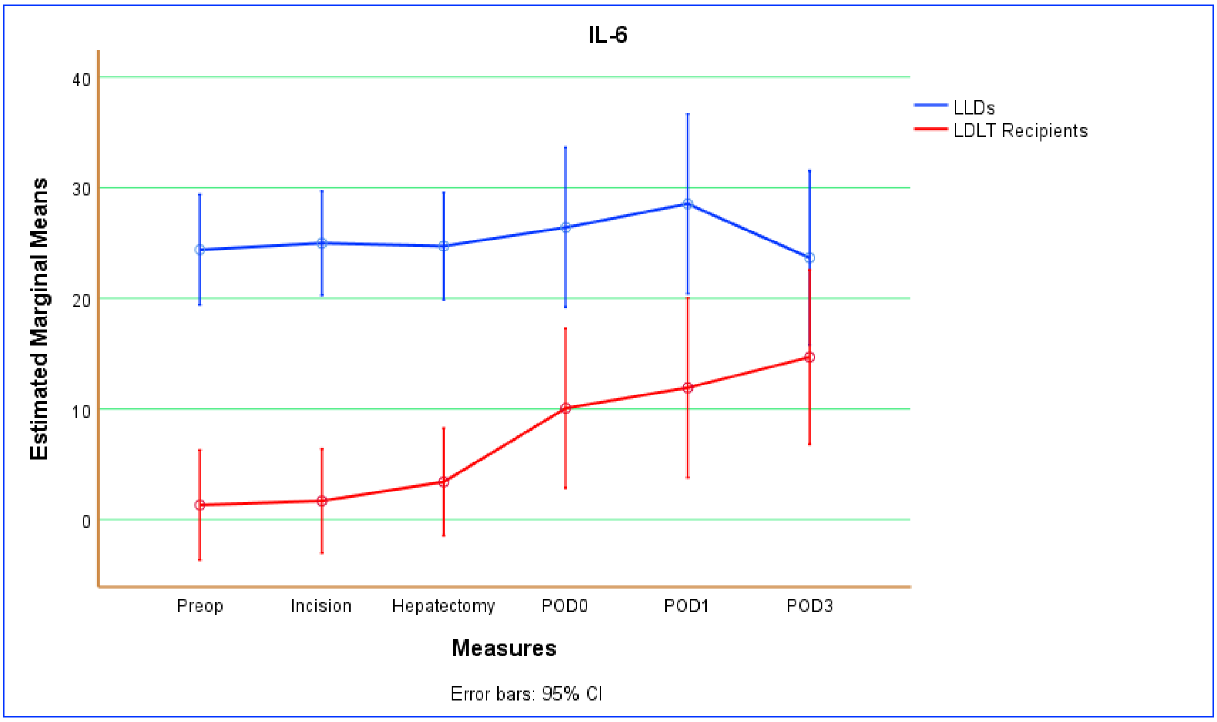

Supplementary Figure S3: Temporal TNF-α Response in LLDs and LDLT Recipients Across Six Perioperative Time Points ($F = 15.3$, $p < 0.001$)

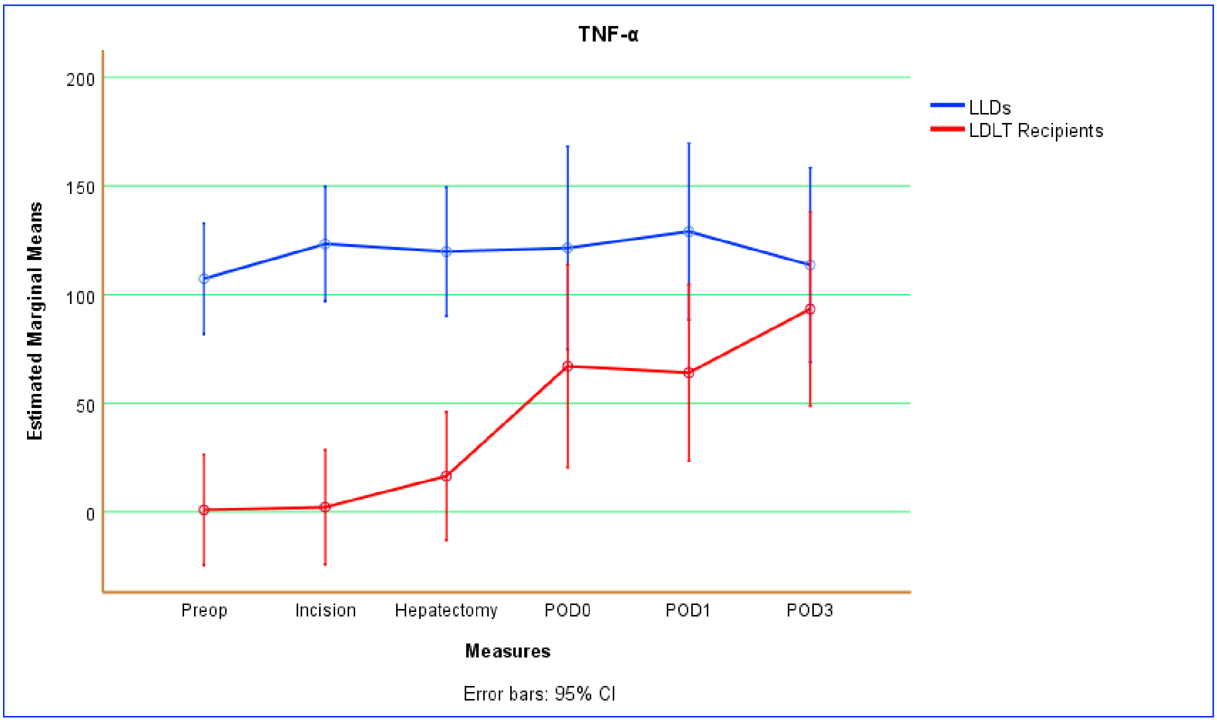

Supplementary Figure S4: Temporal IFN-ɣ Response in LLDs and LDLT Recipients Across Six Perioperative Time Points ($F = 14.1$, $p = 0.001$)

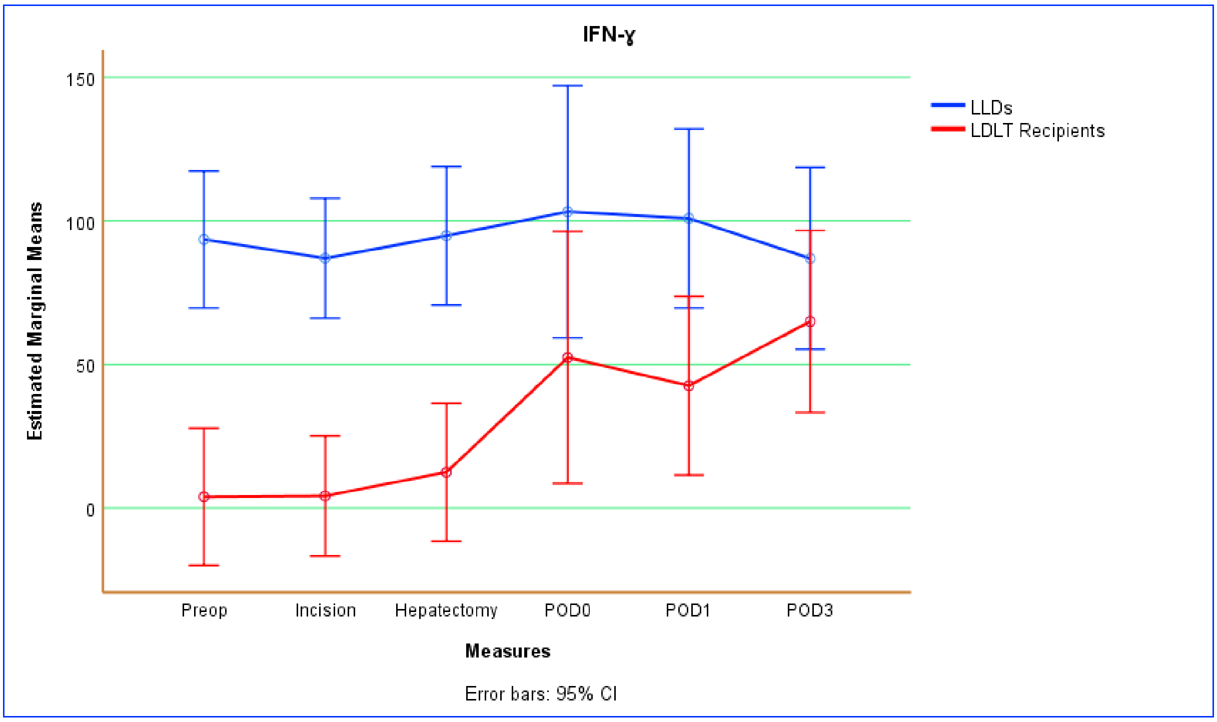

Supplementary Figure S5: Temporal GM-CSF Response in LLDs and LDLT Recipients Across Six Perioperative Time Points (F = 15.4, p< 0.001)

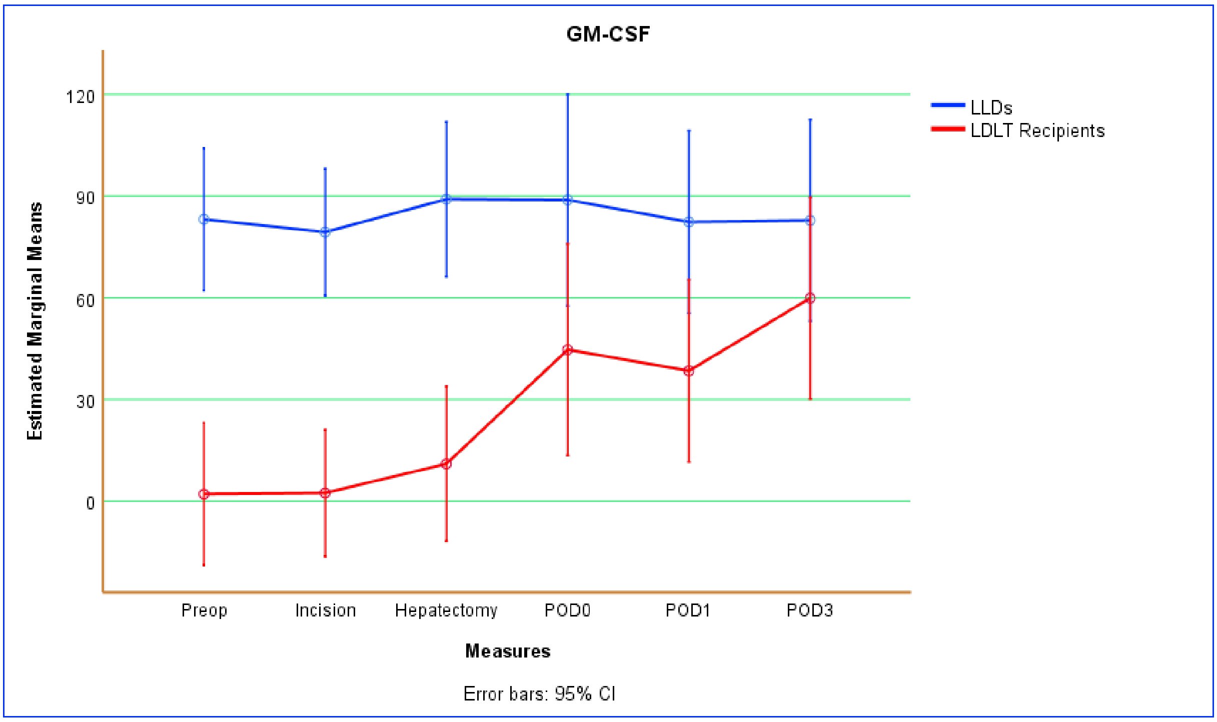

Supplementary Figure S6: Temporal IL-22 Response in LLDs and LDLT Recipients Across Six Perioperative Time Points (F = 18.9, p< 0.001)

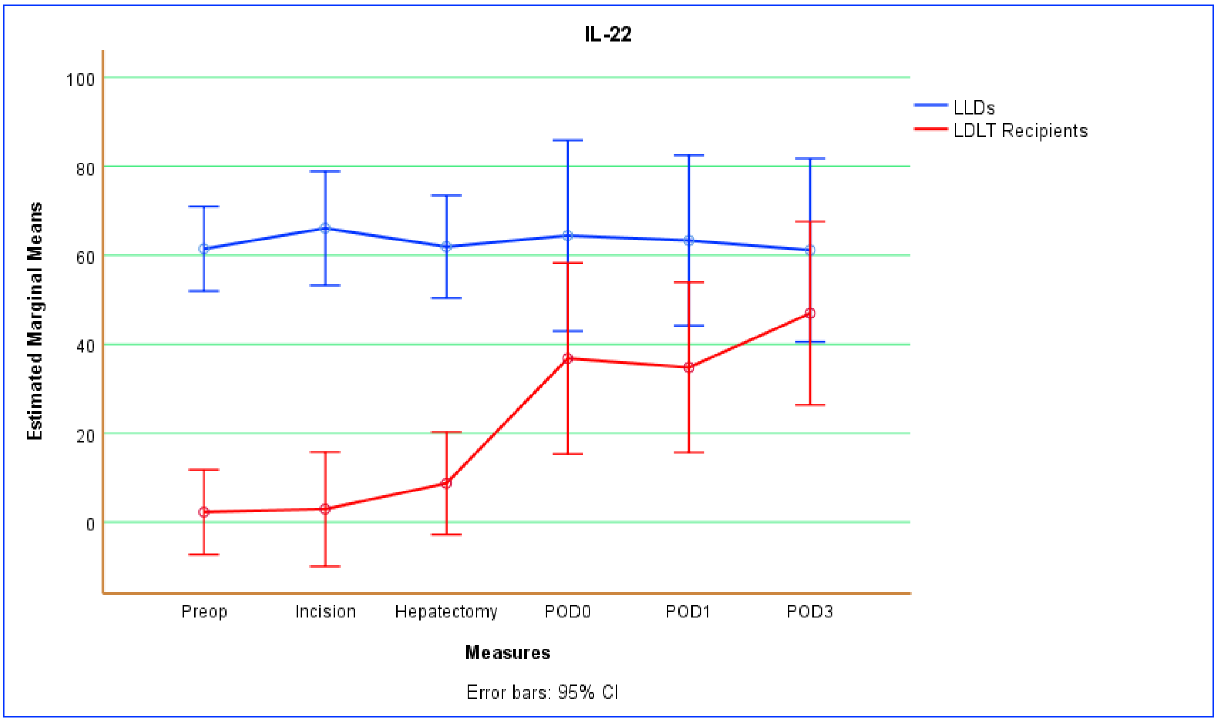

Supplementary Figure S7: Temporal IL-4 Response in LLDs and LDLT Recipients Across Six Perioperative Time Points (F = 29.3, p< 0.001)

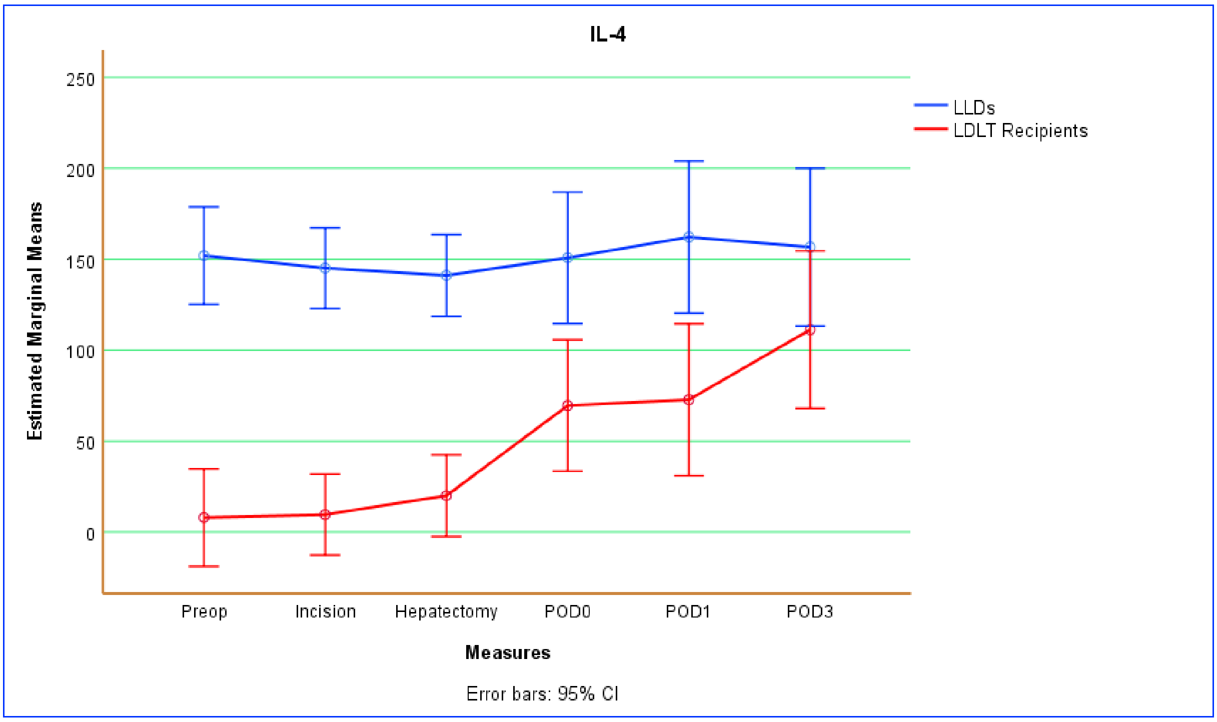

Supplementary Figure S8: Temporal TGF-β Response in LLDs and LDLT Recipients Across Six Perioperative Time Points (F = 23.7, p< 0.001)

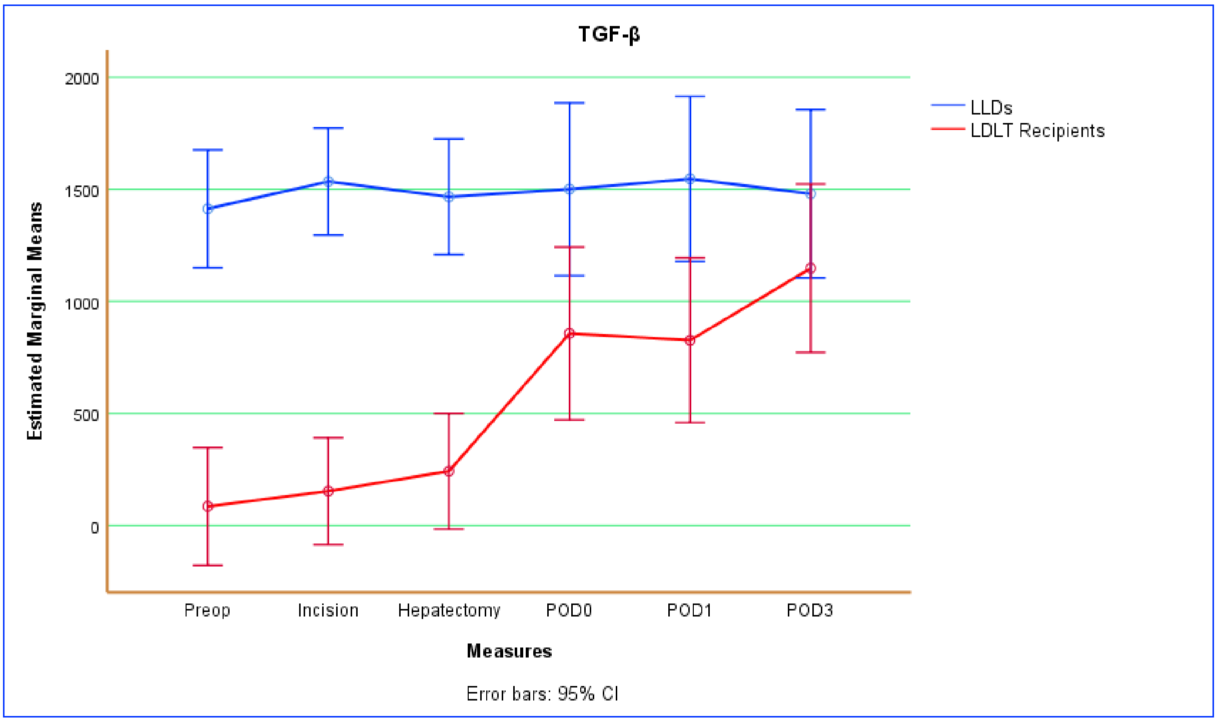

Supplementary Figure S9: Temporal GLDH Response in LLDs and LDLT Recipients Across Six Perioperative Time Points (F = 27.7, p< 0.001)

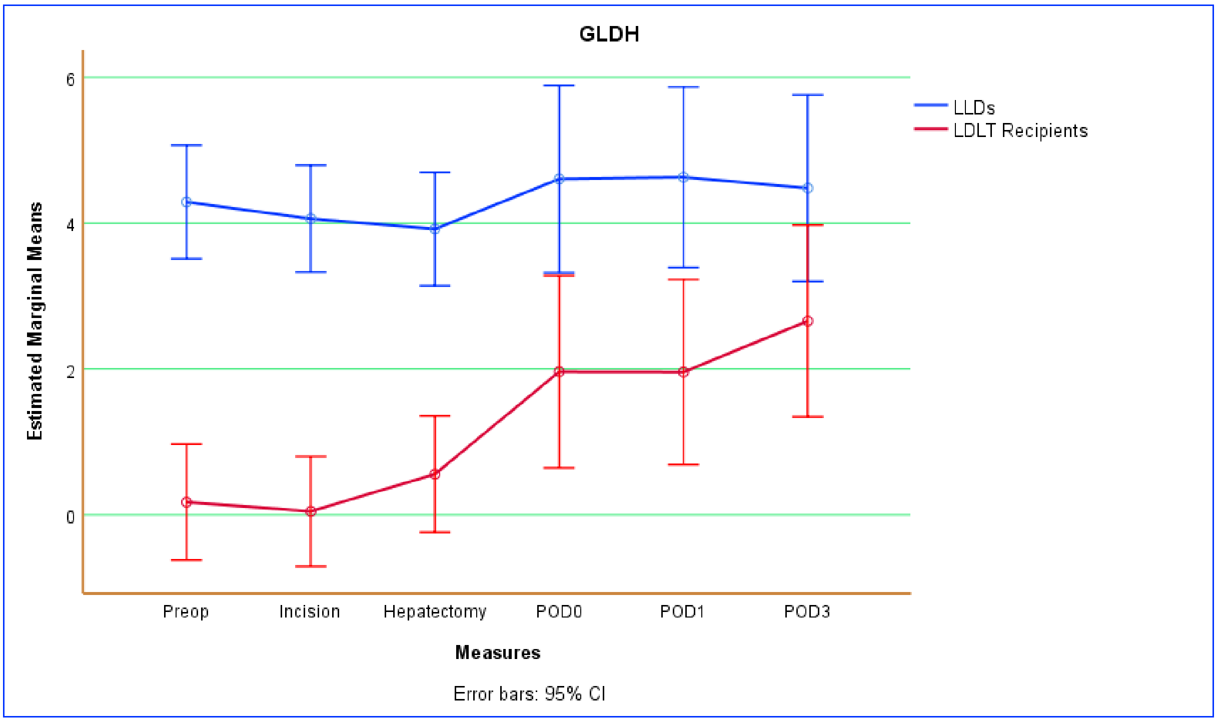

Supplementary Figure S10: Temporal GalactB Response in LLDs and LDLT Recipients Across Six Perioperative Time Points (F = 5.8, p= 0.021)

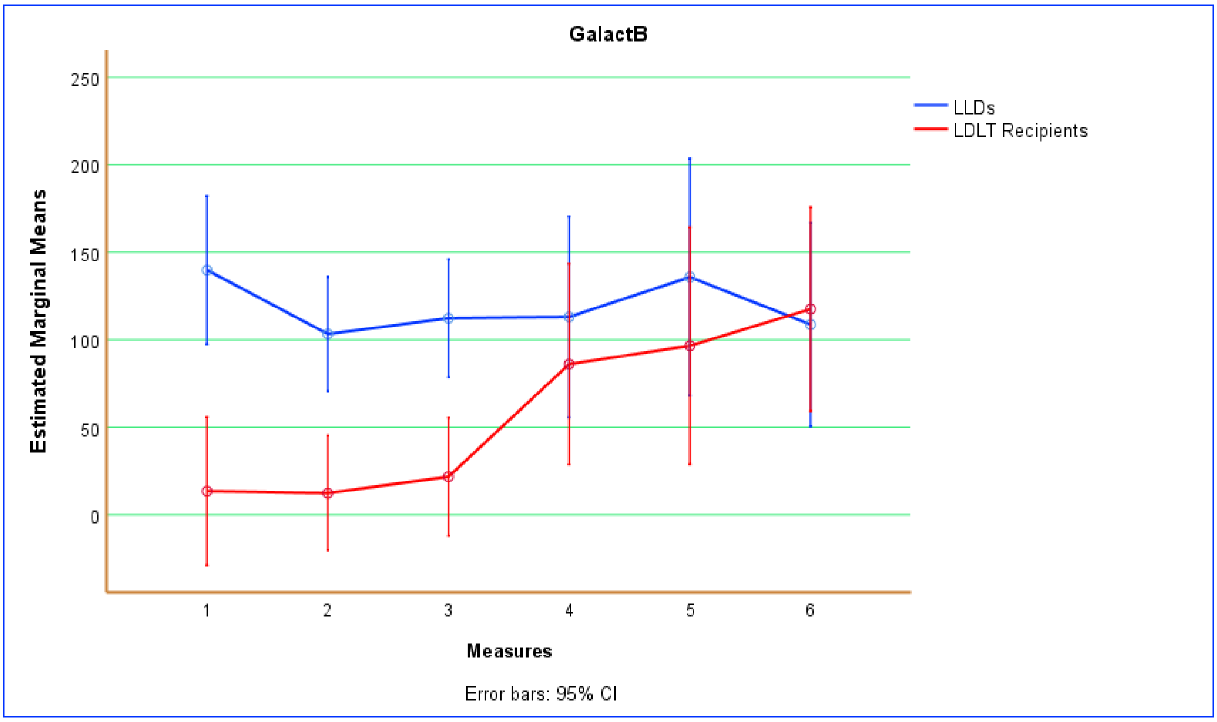

Supplement: Supplementary file 1 [file jcm-14-08970-s001.zip › jcm-3986158-supplementary.pdf]
